# An overview on plasmon-enhanced photoluminescence via metallic nanoantennas

**DOI:** 10.1515/nanoph-2024-0463

**Published:** 2024-11-18

**Authors:** José Luis Montaño-Priede, Mario Zapata-Herrera, Ruben Esteban, Nerea Zabala, Javier Aizpurua

**Affiliations:** Centro de Física de Materiales CFM-MPC (CSIC-UPV/EHU), Paseo Manuel de Lardizabal 5, 20018 Donostia, Spain; Donostia International Physics Center (DIPC), Paseo Manuel de Lardizabal 4, 20018 Donostia, Spain; Department of Electricity and Electronics, FCT-ZTF, UPV-EHU, Bilbao, 48080, Spain; IKERBASQUE, Basque Foundation for Science, María Díaz de Haro 3, 48013 Bilbao, Spain

**Keywords:** plasmonic nanoantennas, photoluminescence enhancement, PLEF, FDTD, quantum emitters

## Abstract

In the realm of nanotechnology, the integration of quantum emitters with plasmonic nanostructures has emerged as an innovative pathway for applications in quantum technologies, sensing, and imaging. This research paper provides a comprehensive exploration of the photoluminescence enhancement induced by the interaction between quantum emitters and tailored nanostructure configurations. Four canonical nanoantennas (spheres, rods, disks, and crescents) are systematically investigated theoretically in three distinct configurations (single, gap, and nanoparticle-on-mirror nanoantennas), as a representative selection of the most fundamental and commonly studied structures and arrangements. A detailed analysis reveals that the rod gap nanoantenna configuration achieves the largest photoluminescence enhancement factor, of up to three orders of magnitude. The study presented here provides insights for the strategic design of plasmonic nanoantennas in the visible and near-IR spectral range, offering a roadmap for these structures to meet specific requirements in plasmon-enhanced fluorescence. Key properties such as the excitation rate, the quantum yield, the enhanced emitted power, or the directionality of the emission are thoroughly reviewed. The results of this overview contribute not only to the fundamental understanding of plasmon-enhanced emission of quantum emitters but also set the basis for the development of advanced nanophotonic devices with enhanced functionalities.

## 1 Introduction

Quantum emitters (QEs), such as semiconductor quantum dots, molecules and vacancies in diamond, have been proposed for a variety of technological applications in different fields, including sensing, imaging, and spectroscopy, due to their ability to absorb and emit photons of specific energy [1], [2], [3], [4], [5], [6], [7], [8]. The emission and absorption of light can be enhanced in the presence of metallic nanostructures that sustain localized surface plasmon polaritons (LSPPs). These plasmonic modes intensify and localize electromagnetic fields within nanoscale volumes, overcoming the diffraction limit of light and enhancing the excitation of QEs strategically placed in the proximity of the metallic surface [9], [10], [11], [12], [13], [14], [15], [16], [17], [18], [19], [20], [21], [22], [23], [24], [25], [26], [27]. Additionally, as predicted by Purcell [28] and theoretically and empirically studied in the last two decades [14], [22], [29], [30], [31], [32], [33], [34], [35], [36], [37], [38], [39], [40], [41], [42], [43], plasmonic modes in metallic nanostructures also accelerate the spontaneous emission rate of QEs, due to the introduction of new radiative and non-radiative decay channels, i.e., an enhanced local density of photonics states. Further, these nanostructures can modify the radiation pattern of the quantum emitter, enabling a more directional emission as compared to that of the isolated QE [44], [45], [46], [47], [48], [49], [50], [51], [52], [53].

Metallic nanostructures are often called nanoantennas due to their capability to modify the absorption and emission of light [49], [54], [55], [56]. This capability is harnessed in a variety of plasmon-enhanced applications including surface-enhanced Raman spectroscopy (SERS) [25], [57], [58], [59], [60], [61], [62], [63], [64], [65], [66], [67], [68], [69], [70] and surface-enhanced infrared absorption (SEIRA) [71], [72], [73], [74], [75], [76], [77]. In this work, we focus on the capability of plasmonic nanostructures to control and enhance photoluminiscence (PL) from quantum emitters [20], [31], [32], [33], [34], [37], [38], [39], [40], [42], [43], [44], [45], [48], [49], [78], [79], [80], [81], [82], [83], [84], [85], [86], [87], [88]. The use of metallic structures to control fluorescence can be traced back to more than two decades ago. Thomas et al. [29] for instance, determined that a system comprising a two-level molecule in proximity to a single silver nanostructure could significantly enhance its photoluminescence by an order of magnitude. The key strategy employed to enhance the QE photoluminescence in these studies is to boost the excitation of the QE while simultaneously maintaining a high radiation efficiency, or radiative yield, defined as the ratio between the radiative and total decay rates [33]. Similarly, control over the emission directionality has been also demonstrated [44], [45], [50], [53].

A successful approach to enhance photoluminescence from QEs consists in positioning it within the plasmonic nanocavity formed between metallic nanostructures, where the electromagnetic field is strongly confined. A straightforward configuration for creating such a plasmonic nanocavity is the dimer (or gap) nanoantenna, composed of two closely located metallic nanostructures [61], [64], [89], [90], [91], [92], [93], [94], [95], [96], [97], [98], [99], [100], [101], [102], [103], [104], [105]. This type of nanoantenna yields significant enhancement of photoluminescence, reaching several orders of magnitude (e.g. 10^3^ in the case of a gold nanorod dimer [43]). A related approach to create a plasmonic nanocavity involves a single nanostructure located above a metallic substrate, creating a nanometric gap separating both structures. This construct is commonly known as nanoparticle-on-mirror (NPoM), and the resulting photoluminiscence enhancement is similar to that observed in gap nanoantennas [17], [22], [40], [106], [107], [108], [109], [110], [111], [112], [113], [114], [115], [116], [117]. Such nanoparticle-on-mirror antennas have been exploited in different applications for optical sensing, imaging, and single photon sources [20], [38], [108], [112], [114], [115], [117], [118], [119], [120], [121].

The advances in nanofabrication have opened new capabilities to develop a wide landscape of nanoantenna configurations that can be employed to enhance photoluminescence from a QE. The performance of each of these configurations strongly depends on a variety of parameters, encompassing aspects such as shape, size, materials, and the relative position of the nanoantennas and QEs [56], [99]. A clear map of general properties of different types of nanoantennas emphasizing their advantages and disadvantages should thus serve to guide effective experimental implementation of optimized plasmon-enhanced fluorescence.

This work provides a comparative overview of the photoluminescence enhancement produced by four canonical gold nanostructures (spheres, rods, disks, and crescents) in three widely-used configurations (single nanoantenna, gap nanoantenna, and nanoparticle-on-mirror). Plasmonic materials like silver and aluminum also offer notable advantages in different spectral regions. Silver, for example, provides a stronger plasmonic response in the visible and near-UV range due to reduced inter-band transitions, though it is more prone to oxidation [122], [123], [124]. Aluminum, commonly used in UV applications, is cost-effective and abundant but experiences higher losses in the visible range [123], [125], [126]. On the other hand, gold is commonly chosen for its superior chemical and thermal stability, along with its effective plasmonic performance in the visible and near-infrared regions, making it a versatile material across a broader range of applications. Thus, we consider the coupling with gold nanostructures. The size of these gold plasmonic antennas is systematically varied within experimentally accessible ranges to tune their optical resonances and maximize light emission from a QE nearby. The resulting structures can be fabricated using well-established techniques such as colloidal chemistry (bottom-up) or lithography (top-down), ensuring nanoantennas of high quality, yield, and reproducibility. Thus, the parameters guiding this investigation, as well as the structures studied, are drawn from the extensive literature on previous experimental works [127], [128], [129], [130], [131], [132], [133], [134], [135].

The aim of this overview is to provide a guide for researchers from various fields to select and design optimal QE – nanoantenna configurations targeting different applications in the visible and near-IR spectral range [51], [53], [136], [137], [138]. We begin by providing a concise overview of the theoretical background, focusing on how to describe the plasmonic enhancement of the photoluminescence signal, and introduce the contributions of the excitation rate and quantum yield to the total power of light emitted. We also briefly detail the numerical methodology applied in our work, based on the use of the finite-difference time-domain (FDTD) method to solve Maxwell’s equations for the near- and far-field calculations. We then outline the four canonical nanostructures and the three configurations considered, discussing the main features of the optical response of each structure. Finally, we present and discuss a comprehensive comparison of the effect of the twelve different plasmonic nanostructures on the excitation enhancement, the decay rates, and quantum yield of the QE, as well as on the resulting photoluminescence enhancement factor (PLEF) and on the directivity of the QE – nanoantenna systems.

## 2 Theoretical background

**Figure 1:**
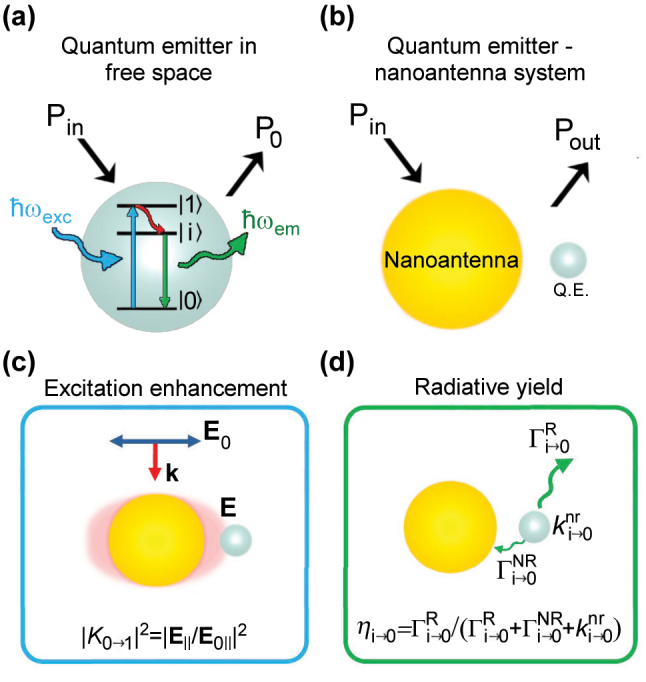
Key magnitudes determining plasmon-enhanced photoluminescence. (a) Energy level diagram of a three-level quantum emitter (QE) with ground (|0⟩), excited (|1⟩), and intermediate fluorescent (|*i*⟩) states. Upon excitation by a photon of energy *ℏω*_exc_ = *ℏω*_0→1_, the QE can emit a photon of energy *ℏω*_em_ ≈ *ℏω*_*i*→0_ after non-radiative relaxation decay from the |1⟩ to the |*i*⟩ state. The power emitted to the far field by the QE in free space is denoted as *P*_0_, and the incident power *P*_in_. (b) Schematic representation of the system consisting on a QE (gray sphere) coupled to a metallic nanoantenna (golden sphere). The power emitted by the QE – nanoantenna system to the far field is labeled as *P*_out_. The power emitted in the presence of the nanoantenna is proportional to two factors represented in (c–d): (c) excitation enhancement |*K*_0→1_|^2^, where |*K*_0→1_| = |**E**_‖_|/|**E**_0‖_| is the electric field enhancement, |**E**_‖_| is the amplitude of the electric field component along the direction of the |0⟩ → |1⟩ transition (evaluated at the location of the QE), and |**E**_0‖_| is the corresponding electric field value of the incident illumination; (d) radiative yield *η*_*i*→0_, defined as the ratio of the radiative decay rate $\Gamma_{i\to 0}^{\text{R}}$ to the total decay rate $\Gamma_{i\to 0}^{\text{T}}=\Gamma_{i\to 0}^{\text{R}}+\Gamma_{i\to 0}^{\text{NR}}+k_{i\to 0}^{\text{nr}}$ of the quantum emitter, where $\Gamma_{i\to 0}^{\text{NR}}$ is the non-radiative decay rate induced by the QE-nanoantenna interaction and $k_{i\to 0}^{\text{nr}}$ is the non-radiative intrinsic decay rate of the QE. The photoluminescence enhancement factor (PLEF) of the QE – nanoantenna system is determined by the ratio *P*_out_/*P*_0_, as defined in Equation (3).

We investigate the enhancement of the photoluminescence emitted by a quantum emitter when it is placed near a metallic nanoantenna. We assume that the QE – nanoantenna system is in the weak coupling regime, i.e., the interaction is not large enough to reach strong coupling and thus it does not trigger phenomena such as vacuum Rabi oscillations or the emergence of hybrid polaritonic modes (at an energy different to that of QE and nanoantenna excitations [139], [140], [141], [142]). However, from the value of the Purcell Factor and of the plasmonic losses computed in this work, it is possible to obtain the coupling strength between a plasmonic mode and a particular QE of a given oscillator strength [143] and thus to estimate the response under strong-coupling conditions [144]. We consider a simplified model of the QE, which is described as a three-level quantum system with ground |0⟩, excited |1⟩ and intermediate fluorescent |*i*⟩ levels (Figure 1a), surrounded by a lossless medium (vacuum for simplicity). The photoluminescence emission from this single QE in vacuum involves three consecutive processes: (i) absorption of a photon of energy *ℏω*_exc_ = *ℏω*_0→1_ that promotes an electron from the ground |0⟩ to the excited |1⟩ state (blue arrow in Figure 1a; *ℏ* reduced Planck constant), (ii) a fast non-radiative relaxation from the excited state |1⟩ to the intermediate state |*i*⟩ (red arrow) and (iii) the electronic decay from the intermediate state |*i*⟩ to the ground state |0⟩ (green arrow) that can result in the emission of a photon. The spectrum of the light emitted within this simplified model adopts the form of a Lorentzian peak with central energy *ℏω*_em_ = *ℏω*_*i*→0_ [24], [36], [145], [146].

We briefly review the established derivation of the equation that describes the photoluminescence signal from such a 3-level QE coupled to the nanoantenna [31], [36]. This derivation considers the population of each level: *N*_0_ for the ground state, *N*_1_ for the excited state, and *N*_*i*_ for the intermediate fluorescent state, with *N*_0_ + *N*_1_ + *N*_*i*_ = 1. The population equation for state |0⟩ is:

(1) $$\frac{dN_{0}}{dt}=-\frac{N_{0}\sigma_{0\to 1}^{\text{abs}}I_{0\to 1}^{\text{loc}}}{\hbar \omega_{0\to 1}}+N_{i}\Gamma_{i\to 0}^{\text{T}},$$

where $\sigma_{0\to 1}^{\text{abs}}$ is the absorption cross-section of the |0⟩ → |1⟩ transition, $I_{0\to 1}^{\text{loc}}$ is the local illumination intensity calculated at the position of the QE, and $\Gamma_{i\to 0}^{\text{T}}$ the total decay rate associated with the |*i*⟩ → |0⟩ transition. The first term in the right-hand side of Equation (1) describes the depopulation of the ground state due to the excitation of the QE to the state |1⟩ by a photon of energy *ℏω*_0→1_. This term is proportional to the population of the ground state (*N*_0_), the absorption cross-section, and the local illumination intensity. Further, the normalization of this term by *ℏω*_0→1_ converts its magnitude from energy to number of excitations (per unit of time). The local intensity is proportional to the square of the electric field enhancement induced by the plasmonic nanoantenna, $I_{0\to 1}^{\text{loc}}=|K_{0\to 1}{|}^{2}I_{0}=|E_{\|}/E_{0\|}{|}^{2}I_{0}$ , with *I*_0_ the intensity of the excitation, *K*_0→1_ the field enhancement, and **E**_‖_ and **E**_0‖_ the component of the electric fields parallel to the dipole orientation of the |0⟩ → |1⟩ transition, with and without antenna, respectively (Figure 1c). For convenience, we will henceforth denote the parallel component of the electric fields as **E** and **E**_0_, without additional notation. The fields are evaluated at the excitation frequency *ω*_0→1_ and position of the QE. This description assumes that the fields are almost constant in all the volume occupied by the (small) QE (point-dipole approximation) [29], [36], [145], [147], which is valid except for extremely localized fields such as in picocavities [115], [148], [149]. Crucially, plasmonic nanoantennas possess the capability to focus the electric field within very confined volumes [13], [26], [49] where |*K*_0→1_|^2^ ≫ 1.

The second term in the right side of Equation (1) captures the relaxation process of the excited quantum emitter from the intermediate |*i*⟩ to the ground |0⟩ state, with energy difference *ℏω*_*i*→0_. This term is directly proportional to the population in the |*i*⟩ state (*N*_*i*_) and to the total decay rate associated with the |*i*⟩ → |0⟩ transition $\left(\Gamma_{i\to 0}^{\text{T}}\right)$ [36]. The term $\Gamma_{i\to 0}^{\text{T}}$ encompasses three distinct contributions (Figure 1d): (i) the radiative decay rate $\left(\Gamma_{i\to 0}^{\text{R}}\right)$ or rate of photon emission to the far field, (ii) the non-radiative decay rate $\left(\Gamma_{i\to 0}^{\text{NR}}\right)$ associated with decay processes where the energy is dissipated by the plasmonic antenna without photon emission (typically as heat), and (iii) the non-radiative intrinsic decay rate $\left(k_{i\to 0}^{\text{nr}}\right)$ due to internal transitions within the QE that are not included explicitly in the simplified level scheme considered here. Thus, $\Gamma_{i\to 0}^{\text{T}}=\Gamma_{i\to 0}^{\text{R}}+\Gamma_{i\to 0}^{\text{NR}}+k_{i\to 0}^{\text{nr}}$ , where only $\Gamma_{i\to 0}^{\text{R}}$ is associated with the emission of a photon of energy *ℏω*_*i*→0_. Equation (1) does not include the (spontaneous or stimulated) decay of the excited state |1⟩ to the ground state because this process is considered to be very slow (compared to that of the |1⟩ → |*i*⟩ transition). This assumption is standard in photoluminescence studies, and is well justified for isolated QEs, but could break for strong plasmonic field enhancement [42], [150], [151].

We always assume weak enough illumination, ensuring that *N*_0_ ≈ 1, so the steady-state solution of Equation (1) is

(2) $$N_{i}=\frac{\sigma_{0\to 1}^{\text{abs}}}{\hbar \omega_{0\to 1}\Gamma_{i\to 0}^{\text{T}}}I_{0\to 1}^{\text{loc}}=\frac{\sigma_{0\to 1}^{\text{abs}}}{\hbar \omega_{0\to 1}\Gamma_{i\to 0}^{\text{T}}}|K_{0\to 1}{|}^{2}I_{0}.$$

Further, the emitted light power *P*_out_ is proportional to the population of the intermediate state *N*_*i*_ and to the radiative emission rate $\Gamma_{i\to 0}^{\text{R}}$ . From Equation (2), *P*_out_ is thus proportional to $|K_{0\to 1}{|}^{2}\Gamma_{i\to 0}^{\text{R}}/\Gamma_{i\to 0}^{\text{T}}=|K_{0\to 1}{|}^{2}\eta_{i\to 0}$ , where $\eta_{i\to 0}=\Gamma_{i\to 0}^{\text{R}}/\Gamma_{i\to 0}^{\text{T}}$ is the radiative yield, a parameter that describes the probability that a QE in state |*i*⟩ results in the emission of a photon, instead of in a non-radiative or internal decay process. Therefore, the photoluminescence enhancement factor of a QE due to the presence of a metallic nanoantenna can be calculated as:

(3) $$\frac{P_{\text{out}}}{P_{0}}=|K_{0\to 1}{|}^{2}\frac{\eta_{i\to 0}}{\eta_{i\to 0}^{0}},$$

where *P*_0_, $\eta_{i\to 0}^{0}=\Gamma_{i\to 0}^{\text{R0}}/\left(\Gamma_{i\to 0}^{\text{R0}}+k_{i\to 0}^{\text{nr}}\right)$ , and $\Gamma_{i\to 0}^{\text{R0}}$ are the emitted power, the intrinsic quantum yield and the radiative decay rate of the isolated quantum emitter in the absence of the nanoantenna (in this situations, the non-radiative decay due to the QE – nanoantenna interaction is zero, $\Gamma_{i\to 0}^{\text{NR0}}=0$ ; the intrinsic decay rate *k* ^nr^ is considered to be unaffected by the antenna) [36], [109]. For simplicity, we are assuming that the decay rates and thus the quantum yield are constant in the spectral width of fluorescent emission.

The usual goal is to use a plasmonic nanoantenna to enhance the PLEF, *P*_out_/*P*_0_ ≫ 1, but this is not always the case. In the following, we identify quenching of the emission with *P*_out_/*P*_0_ < 1, i.e., with emission inhibition [22], [32], [33], [45]. Equation (3) indicates that this enhancement only depends on the near-field enhancement at the illumination frequency *ω*_exc_ (resonant with the |0⟩ → |1⟩ transition) and on the yield at the emission frequency *ω*_em_ (corresponding to |*i*⟩ → |0⟩ transition). We highlight that this description would not be fulfilled under very strong illumination [36], [152]. In the present work, we consider the excitation and emission evaluated at the same frequency, *ω*_exc_ = *ω*_em_, for the sake of simplicity and to provide a clearer interpretation of the underlying principles. However, it is important to note that the field enhancement and quantum yield estimations, as presented here, are also valid to evaluate PLEF in other scenarios where *ω*_exc_ ≠ *ω*_em_.

In the following, to simplify the analysis, we assume that the QE has no intrinsic losses, *k* ^nr^ = 0, so that the intrinsic quantum yield is equal to one, $\eta_{i\to 0}^{0}=1$ . Under this assumption, Equation (3) becomes

(4) $$\frac{P_{\text{out}}}{P_{0}}=|K_{0\to 1}{|}^{2}\eta_{i\to 0}=|K_{0\to 1}{|}^{2}\frac{\Gamma_{i\to 0}^{\text{R}}}{\Gamma_{i\to 0}^{\text{R}}+\Gamma_{i\to 0}^{\text{NR}}},$$

which indicates that any enhancement of the photoluminescence can only be due to an enhancement of the excitation |*K*_0→1_|^2^ > 1, because by definition *η*_*i*→0_ < 1. Using a QE with a low intrinsic quantum yield, $\eta_{i\to 0}^{0}\ll 1$ , can result in much stronger PLEF, as described by Equation (3), but not in a larger total emitted power *P*_out_. Hereafter, $\eta_{i\to 0}^{0}=1$ and we simplify the notation of *K*_0→1_ and *η*_*i*→0_ to *K* and *η*, respectively.

## 3 Calculation method

The PLEF, *P*_out_/*P*_0_, produced by the plasmonic nanoantennas is calculated using electrodynamics simulations based on the finite-difference in time-domain method [153]. We thus neglect quantum effects, such as charge-transfer processes due to electron tunneling across energy barriers [102], [154], [155], which might be relevant in situations involving extremely small (subnanometric) separation distances and that likely result in a decrease of photoluminescence emission due to the quenching of the local electric field as well as the quenching of the molecular transition that occurs when electronic states of the metal and QE hybridize [156], [157]. Following Equation (4), we perform two types of simulations for each configuration, the first one to find the excitation enhancement |*K*|^2^ at the position of the emitter, and the second one to obtain the radiative yield *η* together with the radiative and non-radiative decay rates.

We first consider illumination by a linearly polarized plane wave that excites the bare nanoantenna in the wavelength range 500 nm ≤ *λ*_exc_ ≤ 2,000 nm (with *λ*_exc_ = 2*πc*/*ω*_exc_ and *c* the speed of light in vacuum). The resulting electric field enhancement, |*K*|^2^ = |**E**/**E**_0_|^2^, is obtained by placing an electric-field point monitor at the position of the QE to record the electric field **E**, and using the corresponding **E**_0_ value of the incident illumination to normalize the results. Importantly, this simulation does not include the QE. The polarization and direction of the incidence plane wave, as well as the position where the QE will be placed and orientation of its dipolar transition, are indicated in Section 4.1.

Once the illumination and field enhancement conditions are established, we simulate the emission process by including the QE. In this case, the QE is modelled as a point-like electric dipole source with an emission wavelength in the 500 nm ≤ *λ*_em_ ≤ 2,000 nm range (with *λ*_em_ = 2*πc*/*ω*_em_). We assume the dipole is oriented perpendicular to the nanoantenna surface, as this orientation contributes more significantly to the PLEF compared to when it is oriented parallel, as demonstrated in several studies [24], [30], [31], [36], [42], [44], [158], [159]. The power radiated to the far field of the entire QE – nanoantenna system (*P*_out_) is then obtained by integrating the emitted power over a closed-surface monitor surrounding the system, separated from the source by a distance of twice the maximum wavelength of the dipole source. Simultaneously, within the same simulation, we determine the total power (i.e., including radiative and non-radiative contributions) emitted by the dipole source in the presence of the nanoantenna ( $P_{\text{dip}}^{\text{T}}$ ) by integrating the emitted power over a closed-surface monitor surrounding only the dipole source. Additionally, we simulate the power emitted to the far field by the dipole source in the absence of the nanoantenna $\left(P_{\text{dip}}^{0}\right)$ . The plasmon-induced change of the total Γ^T^, radiative Γ^R^, and non-radiative Γ^NR^ decay rates, as well as the radiative yield *η*, can then be directly related to these values as [159]:

(5) $$\begin{array}{cc} \frac{\Gamma^{\text{T}}}{\Gamma^{0}} & =\frac{P_{\text{dip}}^{\text{T}}}{P_{\text{dip}}^{0}},\\ \frac{\Gamma^{\text{R}}}{\Gamma^{0}} & =\frac{P_{\text{out}}}{P_{\text{dip}}^{0}},\;\text{and}\\ \frac{\Gamma^{\text{NR}}}{\Gamma^{0}} & =\frac{\Gamma^{\text{T}}}{\Gamma^{0}}-\frac{\Gamma^{\text{R}}}{\Gamma^{0}},\\ \eta & =\frac{\Gamma^{\text{R}}}{\Gamma^{\text{T}}}=\frac{P_{\text{out}}}{P_{\text{dip}}^{\text{T}}}, \end{array}$$

where Γ^0^ is the spontaneous decay rate of the QE in the absence of nanoantenna and no intrinsic losses are considered, *k* ^nr^ = 0. The set of Equations (5) shows, that in this case, the quantum yield does not depend on $P_{\text{dip}}^{0}$ .

In addition to enhancing the photoluminescence of the QE, the nanoantenna has also the capability to direct the emitted power towards a preferential direction [44], [45], [46], [47], [48], [49], [50], [51], [52]. This effect can be quantified through the directivity *D* at a particular set of angular coordinates (*θ*, *ϕ*), defined as

(6) $$D\left(\theta ,\phi \right)=\frac{4\pi P\left(\theta ,\phi \right)}{P_{\text{out}}},$$

where *P*(*θ*, *ϕ*) represents the power per unit solid angle emitted in a particular direction (evaluated in the far field), and *θ* and *ϕ* are the polar and azimuthal angles, respectively (*θ* measured from the positive *z* axis of the Cartesian coordinates and *ϕ* measured in the *xy* plane from the positive *x* axis, see Figure S1). Notice that *P*(*θ*, *ϕ*) is normalized by the total radiated power *P*_out_, so that *D* = 1 for an isotropic source. The determination of the power emitted by the different nanoantenna configurations follows the same methodology as in the previous simulations. Specifically, *P*(*θ*, *ϕ*) is calculated over the same closed-surface power monitor surrounding the system, which computes the far-field projection in the direction described by (*θ*, *ϕ*) [160].

In all the simulations, the QE – nanoantenna systems are centered in a cubic simulation domain with perfect matched layers (PML, 60 layers) as boundaries in the three directions. The length of the cubic domain is four times the maximum wavelength considered for the simulations, i.e., 8,000 nm in length. The Courant number, defined as *c*Δ*t*/Δ*x*, where Δ*t* is the time step, and Δ*x* is the spatial step, is set below 0.9 to maintain the stability of the FDTD simulations [153]. In addition, we conduct convergence testing to ensure the accuracy of our findings. The refractive index of gold is taken from Ref. [161], and the medium surrounding the antennas is considered as vacuum.

## 4 Results and discussion

### 4.1 Description of the nanoantennas

**Figure 2:**
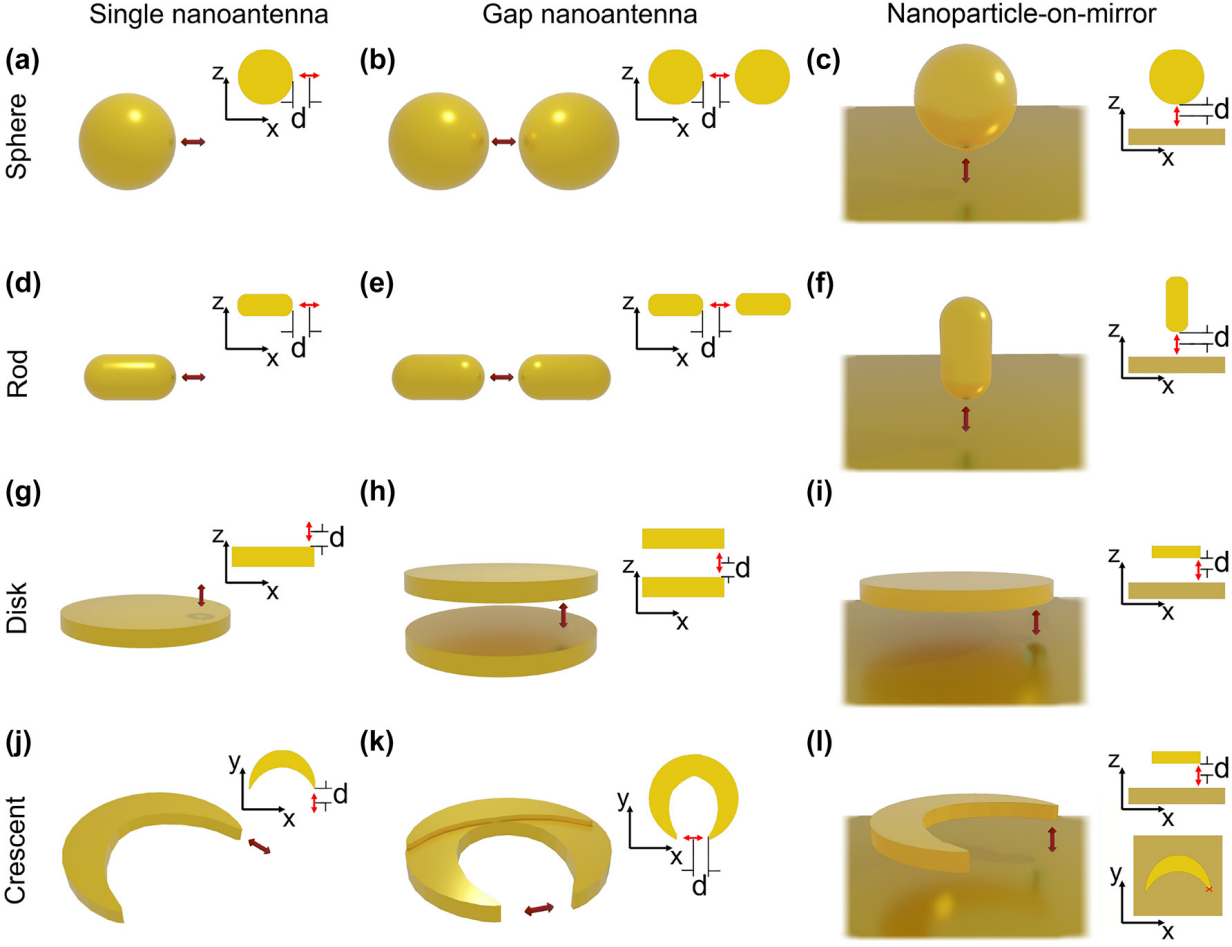
Nanoantennas considered in this work to enhance the photoluminescence of a nearby QE, represented by an electric dipole (red arrow). The canonical nanostructures are: (a–c) spherical particle, (d–f) rod, (g–i) disk, and (j–l) crescent, which adopt three configurations: (a, d, g, j) single, (b, e, h, k) gap, and (c, f, i, l) nanoparticle-on-mirror nanoantennas. The electric dipole, symbolizing the quantum emitter, is located at a distance *d* from the surface of the nanostructures (at the gap center, when a gap is present), with its direction perpendicular to the nanoantenna surface, as indicated in the corresponding right-hand schematics in each panel.

We explore four canonical nanostructures across three distinct antenna configurations, resulting in a total of twelve arrangements (Figure 2). This array of setups is selected to give a general overview of the capacity to enhance photoluminescence from QE – nanoantenna systems that can be fabricated with a high degree of control. First, we select four metallic nanostructures that are commonly studied, synthesized and used in nanophotonics: spherical particle, rod, disk, and crescent nanostructures (Figure 2a, d, g, and j, respectively). The first two, spheres and rods, are nowadays relatively straightforward to fabricate through bottom-up techniques, such as colloidal chemical synthesis, with precise control over their size [127], [131], [132], [134]. Moreover, advances in self-assembly techniques allow for the arrangement of several QE units, and introduce paths towards the placement of QEs at controlled distances from the nanoparticles [43], [162], [163], [164], [165], [166], [167], [168]. The other two selected nanostructures, disks and crescents, can be fabricated with precise control over their shape and size through top-down methods like lithography and evaporation techniques. In these systems, the placement of quantum emitters is facilitated by deposition techniques such as dip coating or chemical self-assembly [169], [170]. Further, while we focus in individual nanoantennas, this approach offers advantages for the fabrication of periodic arrays with different symmetries [128], [129], [130], [133], [135].

We consider three different arrangements of these four canonical nanostructures: single nanoantennas (with the QE in proximity to one isolated nanostructure) [13], [42], [51], [80], [84], [85], [88], [168], [171], gap nanoantennas (or dimers, where two similar nanostructures are separated by a gap with the QE in between) [43], [53], [172], [173], and nanoparticle-on-mirror constructs (where the QE is positioned in the gap between a metallic nanostructure and a metallic substrate that acts as a mirror [22], [40], [113], [133], [174], [175]). All of these configurations have been shown to be well-suited for self-assembly of quantum emitters and to strongly enhance their light emission [32], [37], [40], [43], [51], [53], [86], [99], [116], [130], [137], [176], [177].

In all these systems, the size of these nanostructures plays a crucial role in determining their optical properties and performance in a specified spectral range. We choose the sizes based on the capabilities of fabrication techniques with demonstrated high quality and reproducibility [127], [129], [130], [131], [132], [133], [134], [135]. We consider spherical particles of diameter 20 nm ≤ *D*_Sph_ ≤ 250 nm. The rods have total lengths in the range 25 nm ≤ *L*_Rod_ ≤ 350 nm, with circular cross-section of fixed diameter *D*_Rod_ = 25 nm (that is, the aspect ratio *L*_Rod_/*D*_Rod_ is varied from 1 to 14), and with hemispherical end-caps. The diameter of both the disk and crescent (*D*_Dsk_ and *D*_Crs_, respectively) ranges from 100 nm to 500 nm, with fixed thicknesses of *t*_Dsk_ = *t*_Crs_ = 30 nm. The edges of the disks and crescents form sharp, rectangular angles, with the crescents finished off with slightly curved tips. Additional size specifications for the crescent nanostructures can be found in the Supporting Information (Figure S2, where all geometries are illustrated). Henceforth, for simplicity, we collectively refer to the geometrical parameter that we systematically modify for each nanostructure (*D*_Sph_, *L*_Rod_, *D*_Dsk_, and *D*_Crs_) as their “size”. Other specific references to individual geometrical parameters are made throughout the text as needed.

With regard to the gap nanoantenna and nanoparticle-on-mirror structures, the rods are aligned along the same axis in the gap nanoantenna, and vertically to the substrate in the nanoparticle-on-mirror configuration, which maximizes the electric field enhancement in the gap (Figure 2e and f). We choose the metal-insulator-metal stack configuration for the disk gap nanoantenna, disk-on-mirror and crescent-on-mirror configurations (Figure 2h, i, and l, respectively), as widely explored in the literature [51], [96], [98], [130], [133], [178], [179], [180], [181], [182], [182]. Regarding the crescents gap nanoantenna (Figure 2k), we consider two merged crescent nanostructures, with one of them rotated at an azimuthal angle relative to the other in order to achieve the desired gap aperture (20 nm). Thus, the gap is formed between the tips of each crescent. This distinctive structure is fabricated using a double evaporation process [128], [129].

Additionally, to obtain the PLEF, we simulate the response for two different excitations: an incident light beam (e.g. a laser) modelled as a plane wave, and an emitting dipole representing the quantum emitter. The propagating direction and polarization of the plane wave are selected with the aim at maximizing the enhancement of the electric field by the nanoantennas. Having as a reference the coordinate axis in the schematics on the right-hand side of each of the panels in Figure 2, the plane wave propagates in the −*z* direction for single and gap nanoantennas with the electric field polarization in the *x* direction, and inclined 45° from this direction (in the *xz* plane, with electric field polarized also along this plane) for all nanoparticle-on-mirror nanoantennas.

The distance between the dipole and the nanoantenna surface is fixed at *d* = 10 nm (except when otherwise stated), and the QE is in the middle of the gap for the gap nanoantenna and nanoparticle-on-mirror configurations. This specific distance is selected based on a comprehensive analysis of both theoretical predictions and experimental constraints, as further detailed in Section 4.3. Therefore, the gap size of the gap nanoantenna and nanoparticle-on-mirror is always 20 nm, which is enough to facilitate fabrication and to ensure enough room to locate relatively large emitters (such as quantum dots) at a distance of a few nanometres from the metallic surfaces, and thus to diminish quenching of the emission [32], [33], [45]. As illustrated in Figure 2, the dipole is placed near the tip of the rods, and either directly above or below the flat surface of the disks, in this case near the edge (5 nm lateral displacement towards the center). This lateral position is chosen instead of the center to ensure efficient excitation of the dipolar mode of the individual disks. In the case of the crescents, the emitting dipole is placed in front of the tips (single and gap configurations) or between the tip and substrate. The orientation of the dipole is indicated in Figure 2 and is always chosen to obtain an efficient coupling with the nanoantenna modes (e.g. along the dimer or the particle-substrate axis). For the crescent-gap configuration, a fixed gap size of 20 nm not only ensures large photoluminescence enhancement but also prevents the occurrence of Fano resonansces. These resonances could result from interference between the quadrupolar mode of the crescent and additional dipolar or quadrupolar modes with large charge concentration at the tips when the gap is smaller than 10 nm [183], [184]. By maintaining this gap size, we ensure that these modes do not influence the interaction between the crescent and the emitter.

### 4.2 Plasmonic response of the four canonical nanostructures

**Figure 3:**
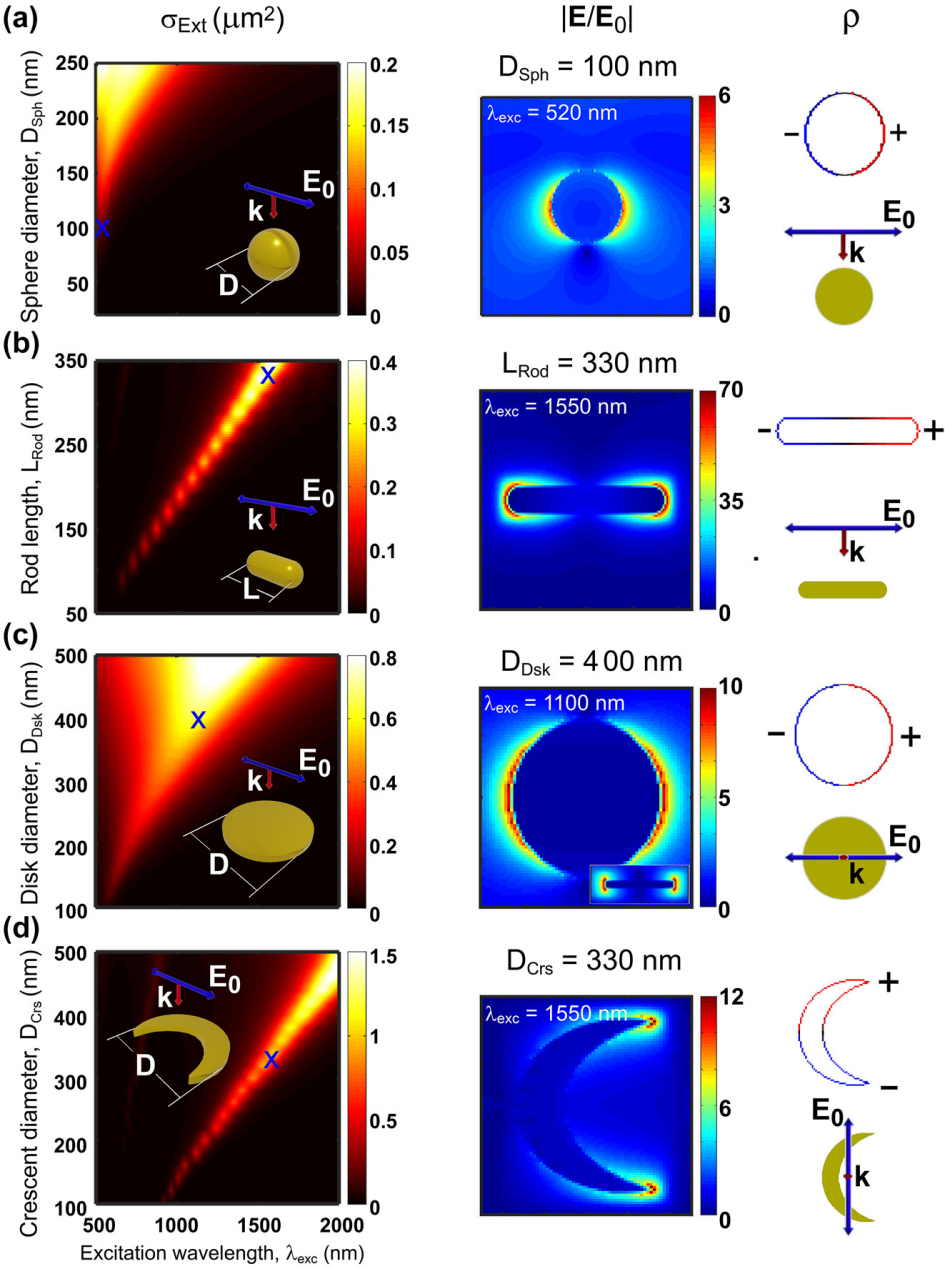
Plasmonic response of the single nanoantennas under plane wave illumination. (Left column panels) Extinction cross-section spectra (*σ*_Ext_) as a function of excitation wavelength and size of the considered single gold nanostructures: (a) sphere of diameter in the range 20 nm ≤ *D*_Sph_ ≤ 250 nm, (b) rod of length in the range 50 nm ≤ *L*_Rod_ ≤ 350 nm (with fixed rod diameter of *D*_Rod_ = 25 nm and hemispherical caps), (c) disk of diameter in the range 100 nm ≤ *D*_Dsk_ ≤ 500 nm, and (d) crescent of diameter in the range 100 nm ≤ *D*_Crs_ ≤ 500 nm. The thicknesses of the disk and crescent are *t*_Dsk_ = *t*_Crs_ = 30 nm. (Central column panels) Spatial distribution of the normalized electric field (|**E**/**E**_0_|) and (right column panels) corresponding surface charge distribution (*ρ*) of the corresponding nanostructures with sizes that yield the maximum electric field at *λ*_exc_ = 520 nm for the spherical nanoparticle, *λ*_exc_ = 1,100 nm for the disk, and *λ*_exc_ = 1,550 nm for the rod and crescent nanostructures, revealing the dipolar nature of the excited nanoantenna mode (blue crosses in *σ*_Ext_ maps indicate the selected excitation wavelengths for visual aid). The sizes in the central and right columns are (a) *D*_Sph_ = 100 nm, (b) *L*_Rod_ = 330 nm, (c) *D*_Dsk_ = 400 nm, and (d) *D*_Crs_ = 330 nm. The antenna geometry as well as the polarization and direction of the incident plane wave are indicated in the insets.

We focus in this section on characterizing the plasmonic modes of the four individual single nanoantennas within the visible-near IR spectral range (500 nm ≤λ≤ 2,000 nm). We calculate the extinction cross-section spectrum (*σ*_Ext_) of these nanoantennas in the selected size range (Figure 3, left panels). In all cases, one dominant resonant band can be appreciated, corresponding to the lowest order mode, which becomes more intense, wider, and redshifts as the nanostructure size increases. This redshift demonstrates the tunability of the nanoantennas response [124], [185]. The resonance occurs at shorter wavelengths for the spherical particle (resonance between ∼520 nm and ∼720 nm in the considered size range), and at much larger wavelengths for the other nanostructures, enabling a resonant response well into the near infrared region. The width of the resonances is particularly large for big spherical and disk nanostructures, and smaller in the other cases. This different behaviour is explained by the larger volume of the former ones, which leads to very strong radiative losses [186], [187], as well as by large gold losses near the surface plasmon at ≈520 nm wavelength [124], [188] (see Figure S6a–c).

To demonstrate the nature of the lowest-order modes of these single nanoantennas, we plot in Figure 3 the spatial distribution of the normalized electric field (|**E**/**E**_0_|, central column panels) and of the corresponding electric surface charge distributions (*ρ*, panels on the right hand side) when the system is excited at the wavelength of the resonant peak. For rods and crescents we chose the size that results in a resonance at wavelength *λ* = 1,550 nm, as this wavelength is particularly important in telecommunications, because it corresponds to the main window of weak absorption in glass fibers. The resonance of spherical and disk nanoantennas does not reach *λ* = 1,550 nm for the simulated sizes, and thus we chose the sizes with resonance at 520 nm and 1,100 nm, respectively. The field and surface charge distribution clearly indicate that the main extinction band corresponds in all the cases to the excitation of the plasmonic dipolar mode. This mode is characterized by a spatial distribution of positive and negative surface charges at opposite sides of the nanoantenna. These charges induce a strong electric field in their proximity, extending away a few tens of nanometers outside the nanoantenna surface, indicating that this specific region is optimal for the excitation of the quantum emitter. The electric dipole is thus placed in this region in the simulations, as described in Section 3.

### 4.3 Effect of QE – nanostructure distance on PLEF

**Figure 4:**
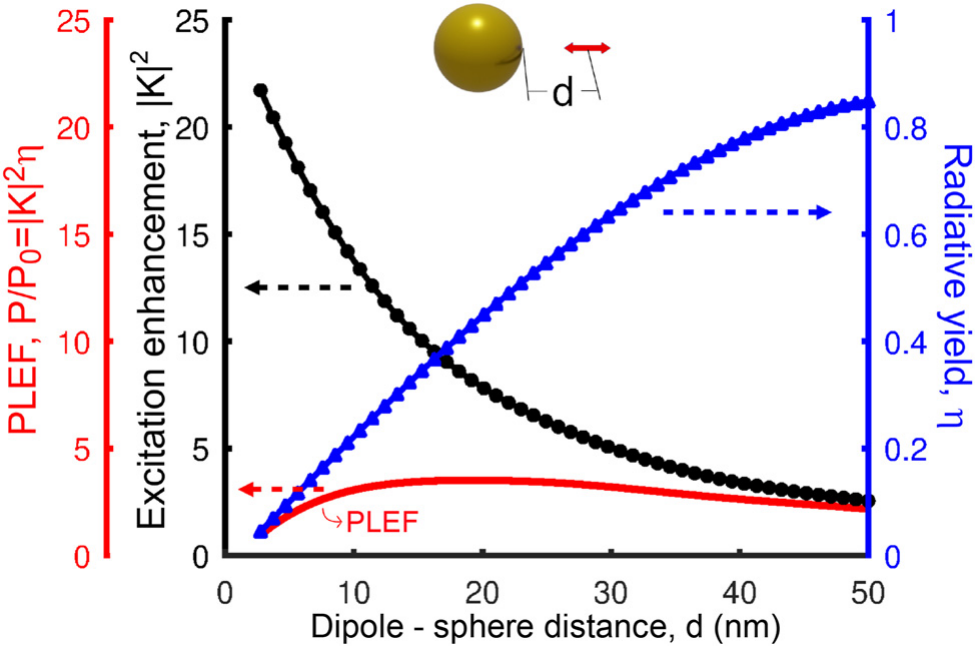
Excitation enhancement (|*K*|^2^, black solid line and circles, left axis), radiative yield (*η*, blue solid line and triangles, right axis), and PLEF (|*K*|^2^ *η*, red solid line, left axis) of the dipole – single spherical nanoantenna. As sketched in the inset, the results are plotted as a function of separation distance (2.5 nm ≤ *d* ≤ 50 nm) between the surface of the sphere and the position of the QE, the latter corresponding to the position where the field is evaluated to obtain |*K*|^2^, or where the dipole is placed to calculate *η*. The diameter of the gold spherical nanostructure is 100 nm and the excitation (*λ*_exc_) and emission (*λ*_em_) wavelengths are both set at 520 nm, the resonant wavelength of the dipolar mode of this nanoantenna.

The separation distance between the QE and the plasmonic nanostructure has a very large impact on the PLEF. To illustrate this effect, we choose as an example a single spherical nanoantenna of diameter *D*_Sph_ = 100 nm. We plot in Figure 4 the excitation enhancement (|*K*|^2^) at the position of the QE under plane-wave illumination, as well as the radiative yield (*η*) characterizing the emission from this QE and the resulting PLEF $\left(P_{\text{out}}/P_{0}=|K{|}^{2}\eta \right)$ . The excitation is tuned to the dipolar mode of the nanoantenna, *λ*_exc_ = 520 nm, and the distance is varied in the range of 2.5 nm ≤ *d* ≤ 50 nm. For simplicity, the difference between excitation and emission wavelength is neglected, i.e., *λ*_exc_ = *λ*_em_ is considered in all the calculations.

The excitation enhancement increases rapidly as the distance decreases (black line and circles in Figure 4), which shows that the plasmons are strongly confined to the region near the nanoantenna. In contrast, the radiative yield approaches unity when the emitter is positioned far from the nanoantenna, but diminishes rapidly at shorter distances (blue line and triangles). This decrease is the consequence of a faster increase of the non-radiative decay rate, compared to the radiative decay rate, as energy from the emitter is transferred to higher order modes of the nanoantenna [33], [44], [79]. As a result, the optimum distance for PLEF (red line) is around *d* = 10 nm for the spherical nanoantenna in Figure 4, as indicated by the red line.

The spherical nanoantenna considered in Figure 4 has the advantage of simplicity, but it is far from optimal to maximize field-enhanced photoluminiscence. Other structures such as rods, as well as gap and nanoparticle-on-mirror nanoantennas, can lead to much stronger field enhancement (Section 4.4) and a slower decrease of the radiative yield with decreasing distance. The latter effect is due to a larger contribution to the decay rate of the lower-order radiative modes in these systems, as compared to that of the higher-order modes. Notably, decreasing the gap size (and thus the QE – nanoantenna distance) in gap and nanoparticle-on-mirror nanoantennas, results in a much faster increase of the field enhancement, as compared to the decrease of the radiative yield, except for very narrow gaps, so that the optimal distance of the emitter to the metallic surface in this type of antennas is significantly smaller, as explicitly shown in Figure S3. We fix the QE distance to 10 nm in the following as a compromise between maximizing PLEF in optimized systems, and facilitating fabrication and ensuring enough room to locate the QEs.

### 4.4 Excitation enhancement

**Figure 5:**
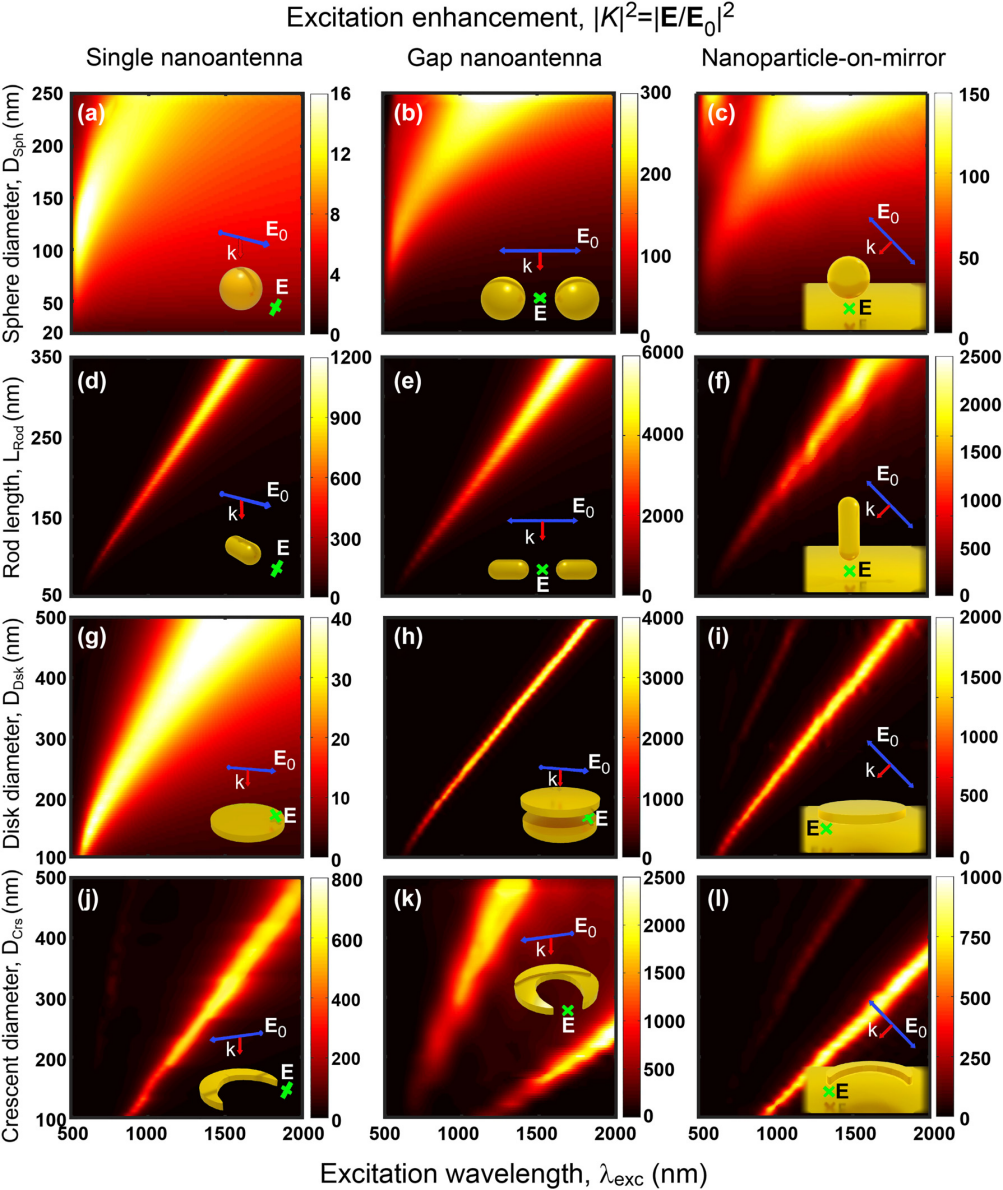
Excitation enhancement maps $\left(|K{|}^{2}=|E/E_{0}{|}^{2}\right)$ of the twelve considered nanoantennas (see insets and Figure 2 as a guide) at the positions where the quantum emitters is placed (green crosses, *d* = 10 nm distance from metal surface). The results are obtained as a function of the nanostructure size (the geometrical parameter under modification is indicated in the y-label) and excitation wavelength (*λ*_exc_). The geometry of the nanoantennas and the polarization of the excitation plane wave are described in Section 4.1.

To analyze more systematically the differences and similarities between all the different systems sketched in Figure 2, we focus first on the excitation enhancement by the incoming plane-wave illumination. Figure 5 shows the calculated maps of the intensity excitation enhancement |*K*|^2^ = |**E**/**E**_0_|^2^ as a function of the excitation wavelength and size of the nanostructures. We evaluate the field at the positions indicated by the crosses in the insets, at 10 nm from the metal surfaces. These positions correspond to the QE locations when calculating the PL, but we emphasize that the QEs are not included in these excitation enhancement calculations.

In general, the excitation enhancement is dominated by the bright lowest-energy mode of each nanoantenna, which redshifts, broadens and induces large field enhancements as the size of the nanoantenna increases. Notably, this mode can be shifted to wavelengths well into the mid-IR spectral range. Plotting the field and charge distribution (see Figure 3d for the crescent, for instance) confirms that the fields are enhanced more strongly at the gaps and/or the region near the tips, so that in all cases the position of the QE (i.e., the position where the fields are evaluated in Figure 5) corresponds to a location of strong field enhancement.

Additionally, a weaker peak at lower wavelengths, corresponding to a higher order mode, can be observed for some of the systems, most prominently for the crescent gap nanoantenna in Figure 5k. The calculated surface charge distribution induced at the crescent gap indicates that this additional mode is quadrupolar (Figure S4). We also note that some modes cannot be excited under normal illumination due to symmetry (subradiant or dark modes) [52], [189], [190], but they can be excited efficiently by a dipole strategically placed close to the nanoantenna (Figures S5 and 6).

Focusing next on the losses, the dominant mode of the sphere gap and sphere-on-mirror nanoantennas inherits the large spectral width of the single sphere dipolar mode, again due to large radiative losses for large diameter and strong gold absorption near *λ*_exc_ ≈ 520 nm. On the other hand, it is more instructive to analyze the mode width of the three disk nanoantenna configurations (Figure 5g–i). The initially broad resonant band in the spectra of the single disk nanoantenna narrows very significantly for both the disk gap and the disk-on-mirror nanoantennas, due to a much weaker radiative nature of the modes excited there. In the case of the disk gap nanoantenna, the incoming light induces a dipole in the bottom disk of opposite direction to that of the top disk (see Figure S7), so that the radiation from both of them partially cancels (i.e., weaker radiation losses) [180] and the quality factor of the resonances (ratio of the resonant frequency to the full-width half maximum) increases. The opposite charges at both disks are also responsible for the intense and strongly confined fields in the inter-disk gap. A similar effect explains the narrow peaks of the disk-on-mirror nanoantenna, where the image charges of opposite sign induced at the substrate play a similar role as those induced at the bottom disk in the gap configuration.

Last, we compare the magnitude of the |*K*|^2^ excitation enhancement for the twelve different systems. All of them can significantly amplify the electric field at the specified QE location, with enhancement factors ranging from ∼10 to over $∼10^{3}$ , potentially reaching up to 6,000 for the rod gap nanoantenna. Single nanoantennas exhibit the smallest enhancement, with the gap nanoantennas consistently performing the best under this criterion. On the other hand, comparing the four canonical nanostructures, the rod stands out for producing the highest field amplification. As a consequence, the rod gap nanoantenna (Figure 5e) can be noted as the most effective one in enhancing the excitation of the quantum emitters. However, many of the other nanoantennas also result in very large values of |*K*|^2^ at resonance, with |*K*|^2^ > 100 for all systems at the optimal size, except for the single spherical and the single disk nanoantennas. This diversity provides researchers with a versatile range of options to select the nanoantenna that most effectively meets the requirements and overcomes the practical limitations of a specific application.

### 4.5 Radiative yield of the QE – nanoantenna systems

**Figure 6:**
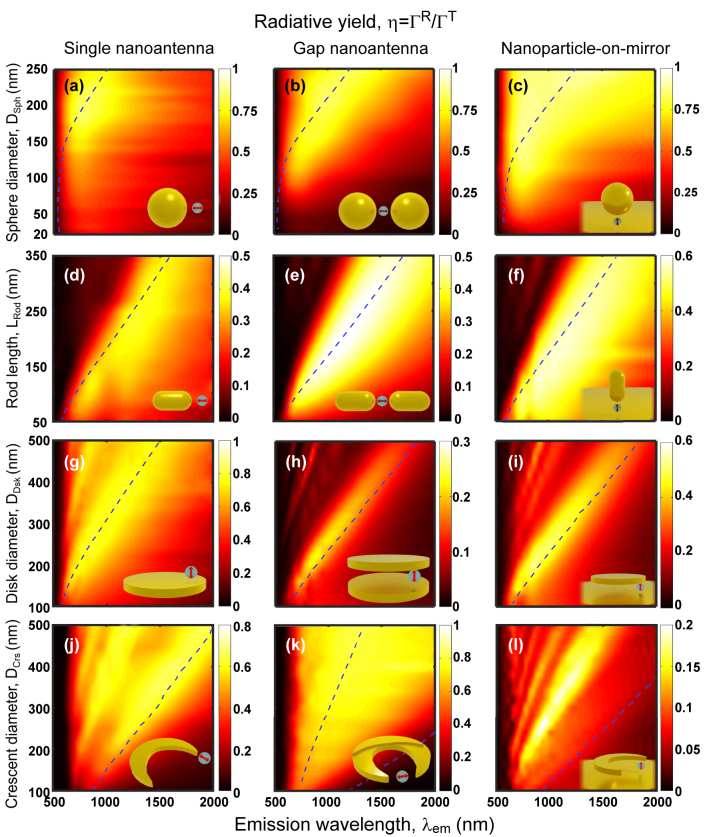
Maps of radiative yield (*η* = Γ^R^/Γ^T^) of the dipole – nanoantenna systems in the corresponding insets (also Figure 2) calculated for *d* = 10 nm dipole-nanoantenna distance and no intrinsic losses (*k* ^NR^ = 0). The yield is obtained as a function of the nanostructure size (the geometrical parameter changed is indicated in the *y*-label) and emission wavelength (*λ*_em_). The point-like dipole that excites the nanoantennas is enclosed by a gray sphere in the insets to distinguish it from the background and to represent the finite physical size of the QE. The geometry of the nanostructures and the dipolar excitation are described in Section 4.1. Blue dashed lines indicate the main plasmon resonant positions of the nanoantennas.

To analyze the emission process, the crucial parameter in typical photoluminescence experiments is the radiative yield, *η* = Γ^R^/(Γ^R^ + Γ^NR^), as given by the radiative (Γ^R^) and non-radiative (Γ^NR^) decay rates of the quantum emitter in close proximity of the plasmonic nanoantenna (Section 2 and Equation (4), obtained for no intrinsic losses). The behavior of Γ^R^ and Γ^NR^ is described in Supporting Information (Figures S5 and S6, respectively). In this section, we analyze the dependence of the radiative yield on wavelength and size of the nanoantennas for all the systems considered here. The results (see Figure 6) are obtained by solving the response of the system when illuminated by a dipole source at *d* = 10 nm from the nanostructure surface, following the methodology detailed in Section 3. For reference, the plasmonic resonant positions (extracted from Figure 5) are indicated in Figure 6 with blue dashed lines, which show that one needs to be careful when interpreting the radiative yield, because the maxima of *η* do not necessarily correspond to the plasmonic resonances.

In most cases, the radiative yield at the resonant frequencies associated with large field enhancement (Figure 5) falls within the *η* ≈ 0.4 − 0.8 range. These results show that absorption in the metal generally leads to a moderate decrease of the radiative yield at resonance, much weaker than the enhancement of the excitation studied in Figure 5. The consequence is an increase of the photoluminescence signal, as shown in the next section. Further, it is worth noting that we consider in this work a QE with no intrinsic losses *k* ^NR^ = 0, but when *k* ^NR^ is large the intrinsic yield of the isolated QE is small, and thus the plasmonic response can actually increase the yield [109].

By comparing the different systems, one can observe that spherical nanoantennas of diameter exceeding 100–150 nm demonstrate particularly large radiative yield (Figure 6a–c; note that the colorbar changes from panel to panel). We attribute this finding to the very large volume of these structures, which increases radiation efficiency. The strong dependence of radiation efficiency on size also explains the weaker yield that is typically found as the size of the antennas is reduced to very small sizes in Figure 6. To further stress this connection between nanoantenna volume and radiative yield, we note as an example that a single sphere of 60 nm diameter and a single rod of 250 nm in length (*D*_Rod_ = 25 nm), both of approximately the same volume, present a similar yield at the dipolar resonance, *η* ≈ 0.4. This analysis emphasizes the importance of taking into account both the nanoantenna geometry and aspect ratio in the pursuit of optimizing radiative yield.

On the other hand, the radiative yield at resonance can be comparatively low for the disk gap and crescent-on-mirror nanoantennas (Figure 6h and l), despite a relatively large volume. This low *η* is a consequence of the destructive interference of the emission from the dipoles of opposite orientation excited at each of the two disks, or at the nanostructure and the substrate. The radiative yield can be increased in these structures, for example, by the use of a nanopillar [191].

For completeness, the maps of radiative Γ^R^/Γ^0^ and non-radiative Γ^NR^/Γ^0^ decay rate enhancements are shown in the Supporting Information, in Figure S5 and S6, respectively. The radiative rate follows rather closely the behavior of the square of the field enhancement |*K*|^2^ in Figure 5 due to the reciprocity principle [49], [56], [192]. The non-radiative rate of the systems involving the rods, disks and crescents show similar resonant bands as the radiative rate and the field enhancement, but higher-order modes can be more prominent due to their larger associated losses. In contrast, no dipolar-mode band is observed in the non-radiative rate contribution when the QE is near a spherical nanoparticle because the coupling with the high order modes (or, equivalently, with the pseudomode [68], [193]) dominates the interaction in that case.

### 4.6 Photoluminescence enhancement factor of the canonical QE – nanoantenna configurations

**Figure 7:**
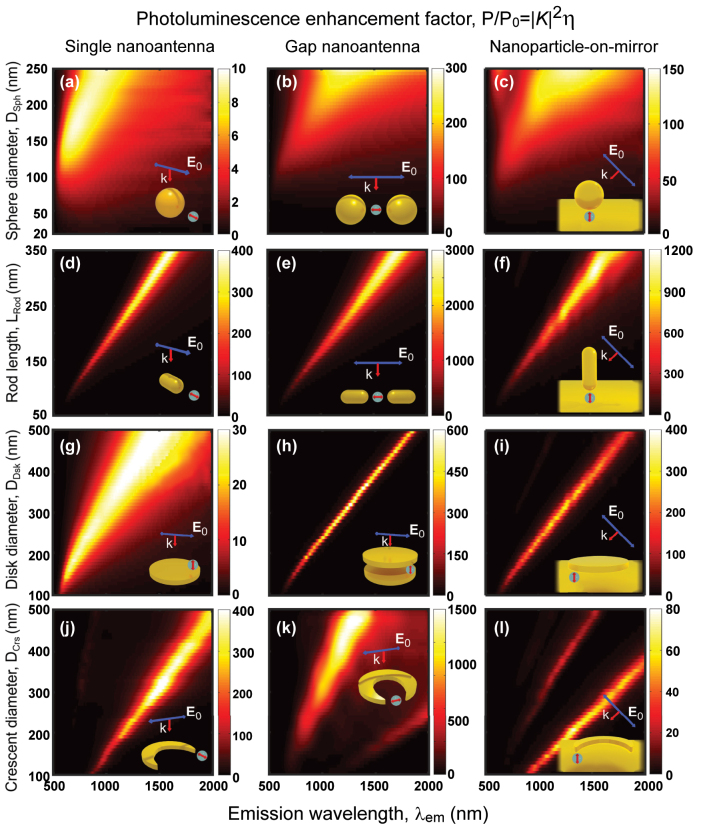
PLEF maps (*P*_out_/*P*_0_ = |*K*|^2^ *η*/*η*_0_) of the dipole – nanoantenna systems in the insets (also Figure 2) as a function of the nanostructure size and emission wavelength (*λ*_em_). The dipole that excites the system is placed at *d* = 10 nm from the nanostructure and has no intrinsic losses (*k* ^NR^ = 0). The geometry of the nanostructures and the polarization of the excitation plane wave are defined in Section 4.1. These results are obtained by multiplying the excitation enhancement (Figure 5) by the radiative yield (Figure 6), considering *λ*_em_ = *λ*_exc_. The point-like dipole is enclosed by a gray sphere in the insets to distinguish it from the background.

The enhancement of the photoluminescence emitted by QEs coupled to nanoantennas is governed by the combined effects of the excitation enhancement |*K*|^2^ = |**E**/**E**_0_|^2^ at the excitation wavelength (Figure 5) and the radiative yield *η* = Γ^R^/Γ^T^ at the emission wavelength (Figure 6). This relationship is encapsulated in the expression *P*_out_/*P*_0_ = |*K*|^2^ *η*, derived in Section 2 for a simple situation with no intrinsic losses of the QE (Equation (4)). Figure 7 showcases the computed PLEFs for all the canonical dipole – nanoantenna systems studied here, considering *λ*_exc_ = *λ*_em_ for simplicity.

A large PLEF is observed across the twelve nanoantennas under consideration, ranging from 10 to 10^3^. The single spherical nanoantenna demonstrates the least favorable enhancement, achieving up to over 10 times. In contrast, the rod nanostructure stands out as the choice of maximum potential enhancement, reaching up to 400 times for the single configuration, 3,000 times for the gap configuration, and 1,200 times for the nanoparticle-on-mirror configuration. Figure S8 in the Supporting Information shows that the orientation of the rod is critical in the nanoparticle-on-mirror configuration, as much smaller enhancements are obtained for horizontally oriented rods. Only the single crescent nanoantenna equals the PLEF of the single rod counterpart. It is worth mentioning that the crescent gap nanoantenna can achieve PLEF as high as 6 × 10^3^ in the mid-IR range (2,500 nm ≤ *λ* ≤ 4,000 nm) for the considered size range (Figure S9). The maps obtained resemble visually those representing the excitation enhancements displayed in Figure 5, as both show clear bands associated with the excitation of the resonant modes. However, both excitation enhancement and radiative yield need to be considered for a detailed analysis. For example, in small nanoantennas, the PLEF can decrease much faster for decreasing size than one could expect from the excitation enhancement, as a consequence of a decreasing radiative yield. This effect can be appreciated more directly in the single spherical and sphere-on-mirror nanoantennas in Figure 7a and c, respectively. As a further example, the crescent-on-mirror nanoantenna induces a five-fold higher |*K*|^2^ excitation enhancement compared to the sphere-on-mirror nanoantenna, but the radiative yield at resonance of the latter (*η* ≈ 0.75) significantly exceeds that of the former (*η* ≈ 0.075). As a consequence, the sphere-on-mirror nanoantenna can enhance the photoluminescence twofold more than the crescent-on-mirror nanoantenna.

**Figure 8:**
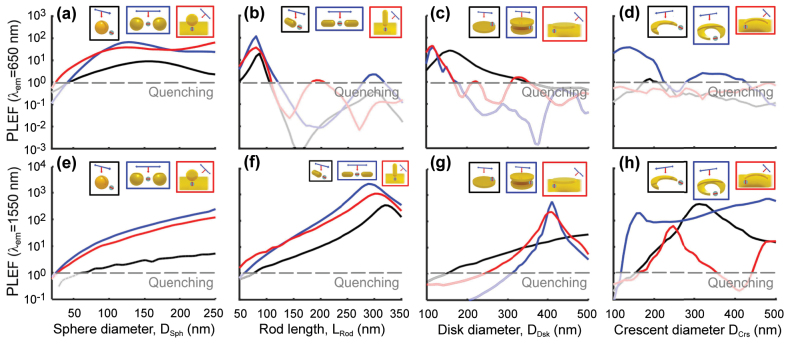
PLEF at *λ*_em_ = 650 nm (top panels) and *λ*_em_ = 1,550 nm (bottom panels) of the dipole – nanoantenna systems in the insets (also Figure 2), as a function of the nanostructure size for (a, e) sphere, (b, f) rod, (c, g) disk, and (d, h) crescent nanostructures with single (black line), gap (blue line), and nanoparticle-on-mirror (red line) nanoantennas. The distance of the dipole from the nanoantenna is *d* = 10 nm. These results correspond to vertical cuts in Figure 7. A gray dashed line serves as a reference, indicating the threshold below which photoluminescence is quenched, i.e., where the decrease in quantum yield outweighs the increase in excitation enhancement resulting in PLEF <1 .

We highlight that large PLEF >25 can be obtained in all possible configurations except for the single spherical nanoantenna, and even stronger values of up to 14,000 can be obtained by reducing the gaps size, as illustrated in Figure S10 in the Supporting Information. Notice that, although Figure 7 considers a large range of emission wavelengths and nanoantenna sizes, the emission (and excitation) wavelengths are fixed in many typical experimental realizations. We consider this situation explicitly by selecting two representative wavelengths, (i) *λ*_em_ = *λ*_exc_ = 650 nm, a value in the visible range that has been used in laser diodes technology [194], [195], [196], and where convenient quantum emitters can be found, and (ii) *λ*_em_ = *λ*_exc_ = 1,550 nm, an infrared wavelength that is standard in silica fiber-based communication technology [147]. Figure 8a–h compare the PLEFs of all the twelve dipole – nanoantenna systems as a function of the size of the nanoantennas for these two wavelengths (corresponding to vertical cuts in Figure 7).

We first focus on the emission at visible wavelengths, *λ*_em_ = 650 nm. Many systems display comparable enhancement, PLEF $\approx 10‐10^{2}$ , at this wavelength for the optimal sizes. For example, the rod – gap nanoantenna (which has the advantage of requiring relatively little gold material for fabrication owing to its small volume [43]) presents a maximum PLEF ≈110 .

Alternatively, systems that comprise spherical nanoparticles show a very broad maximum due to the low quality factor of the resonance, but significant PLEF in the visible range, up to 10^2^, can still be reached for large diameter and for the gap or nanoparticle-on-mirror nanoantennas (Figure 8a), while photoluminescence is strongly quenched for small diameters, i.e., *P*_out_/*P*_0_ < 1. Only the single crescent and crescent-on-mirror nanoantennas quench the photoluminiscence, or result in an extremely weak enhancement (PLEF ≲ 1) for all sizes considered (Figure 8d). The reason is that the higher modes supported by these systems at *λ*_em_ = 650 nm are very weakly radiative, not coupling efficiently with far-field radiation.

Last, we examine in Figure 8e–h the PLEF in the near-infrared *λ*_em_ = 1,550 nm wavelength. The maximum enhancement achieved at the optimal size is generally larger than for the visible *λ*_em_ = 650 nm wavelength. Once again, rod nanoantennas outperform others in enhancing the PL, but many other configurations enable enhancements in the range PLEF $\approx 10^{1}-10^{3}$ .

In practical applications, plasmonic nanoantennas are often integrated with dielectric substrates or placed within surrounding media, both of which can influence PL enhancement effects. A substrate or a medium with a higher refractive index can induce a redshift in the plasmon resonance and alter the local electric field distribution near the nanostructures, with part of the electric field tending to be redirected toward the substrate, which can reduce the intensity of the field available to interact with the QE [49], [56], [171], [197], [198], [199]. Nevertheless, the main objective of our study is to provide a comprehensive guide and a strong foundation for understanding the intrinsic behavior of plasmonic nanoantennas. While substrates or surrounding media may cause a resonance shift, the core principles and findings remain applicable. A simple adjustment for the redshift would effectively account for these environmental effects without diminishing the relevance of the insights described here.

In this context, significant fluorescence enhancement has been documented in various experimental studies, indicating that our predictions are consistent with real-experimental observations. For instance, Orrit et al. observed an enhancement factor of up to 1,100-fold in gold nanorods, primarily due to the proximity of the emitter to the regions of maximum electric field enhancement of the rods [88]. Similar enhancements were reported in polymer-coated gold nanorods [200] and shell-isolated nanoparticles (SHINs) [201]. Furthermore, studies utilizing DNA origami for the arrangement of nanoparticles have shown a maximum enhancement of two or even three order of magnitude [37], [43], affirming the critical role of emitter positioning. Additionally, various nanoparticle-on-mirror configurations exhibit enhancement factors ranging from tens to thousands [175]. These experimental findings corroborate the practical relevance of our theoretical predictions as a robust framework for guiding future experimental endeavors in plasmonic nanoantenna research.

### 4.7 Directivity

**Figure 9:**
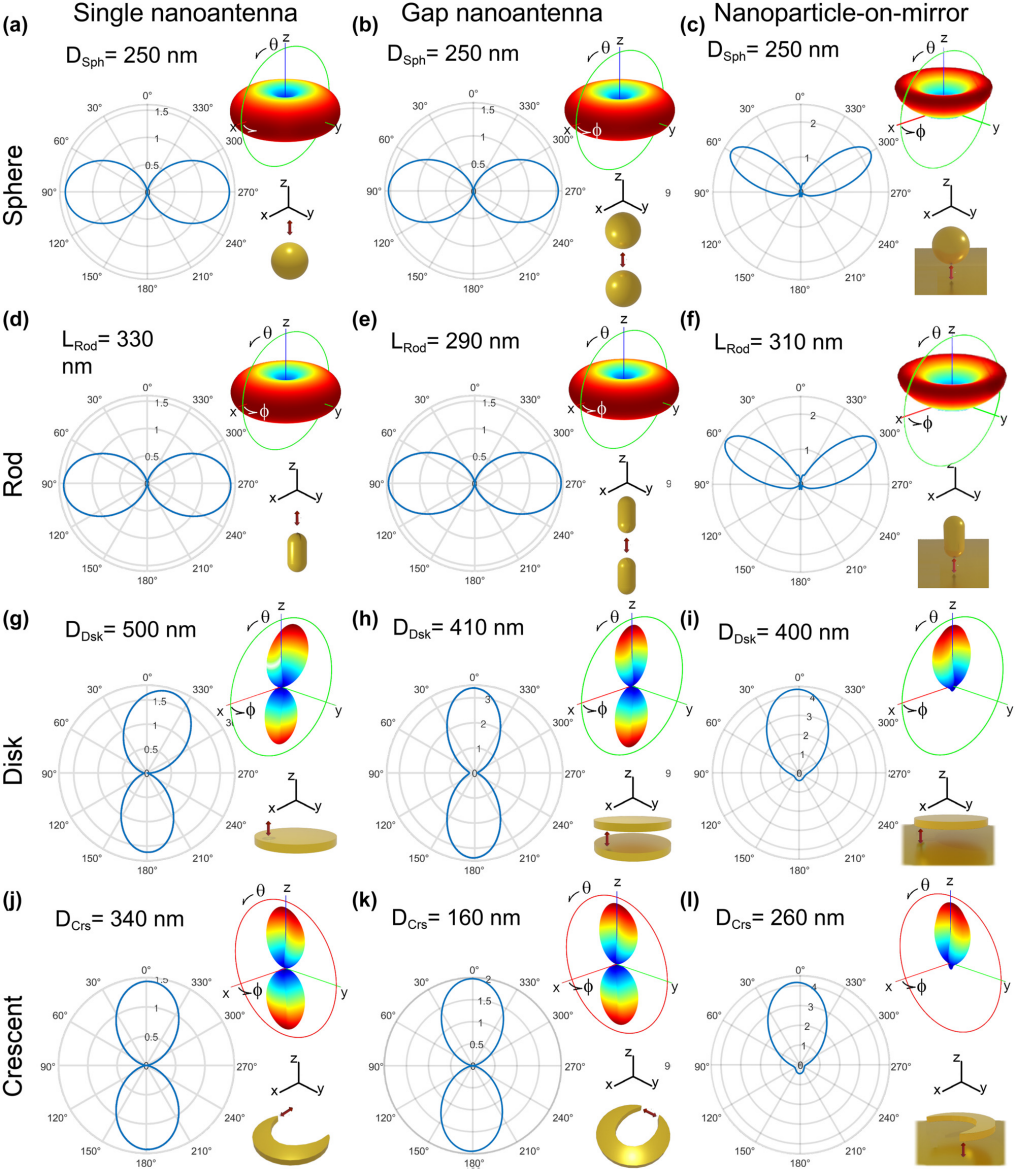
Polar plots of the directivity of the dipole – nanoantenna systems displayed in the bottom right insets of each panel (also Figure 2). In all cases, 3D polar plots as a function of the polar (*θ*) and azimuthal (*ϕ*) angles are shown on the top-right inset and a 2D cut is shown on the left. The full 3D directivity is represented with color gradient ranging from blue, representing the minimum value (0), to red, signifying the maximum directivity. The 2D-polar plots are displayed along the green or red circle in the top right insets, the former included in the *xz* plane and the latter in the *yz* plane, according to the coordinate axis also shown in the figures. The sizes of the nanostructures correspond to those that maximize the PLEF at the photon emission wavelength *λ*_em_ = 1,550 nm.

Another significant aspect of the light emitted by QE – nanoantenna systems is its directivity (Equation (6)), which indicates the extent to which the nanoantennas emit selectively towards a given direction. The top right insets in Figure 9 illustrate the directivity for the twelve dipole – nanoantenna systems through 3D polar plots as a function of the polar (*θ*) and azimuthal (*ϕ*) angles. The plots of the left side display 2D cuts at relevant planes. The directivity is shown for selected nanoantenna sizes that yield maximum PLEF at *λ*_em_ = 1,550 nm (Figure 8).

In general, the directivity pattern and its maximum value vary strongly from system to system, but the values remain relatively modest, which is a direct consequence of the reduced size of the nanoantennas [202]. The systems based on spherical nanoparticles (Figure 9a–c) or rods (Figure 9d–f) exhibit an axisymmetric directivity pattern with respect to the *z* axis (see coordinate axis in the figure), inherited from the common rotational symmetry of the system and illumination. On the other hand, the directivity patterns of the systems based on disks (Figure 9g–i) and crescents (Figure 9j–l) are non-axisymmetric, because the symmetry is broken by the nanostructures or because the dipole is moved away from the symmetry axis of the nanoantenna.

We first focus on the single and gap nanoantenna configurations (i.e., no gold substrate), where two different types of emission patterns can be observed. On the one hand, the systems using spherical nanoparticles and rods present the characteristic emission of a dipole oriented along the *z* axis (corresponding to the orientation of the dipole and the symmetry axis of the antennas), with preferential emission towards the perpendicular *xy* plane. In contrast, for disks and crescents, the emission is preferentially in the *z* direction, also when the dipole is oriented in this same direction. This difference highlights that, when an emitter couples to a nanoantenna, it is the properties of the excited plasmonic modes that often determine the directivity pattern [50], [52], [53]. More complex directivity patterns are possible by coupling the quantum emitter with higher order modes of the systems studied [40], [51].

Furthermore, the substrate has a strong effect on the directivity, blocking the direction in the downwards direction. In the case of crescent-on-mirror and disk-on-mirror nanoantennas, the emission pattern in the upward direction is very similar to that of the gap disk and gap crescent antennas. On the other hand, the sphere-on-mirror and rod-on-mirror antennas emit preferentially towards a *θ* = 60° angle with respect to the *z* axis, instead of *θ* = 90° for the equivalent systems without a substrate.

We last note that, according to the reciprocity principle [49], [56], [192], any nanoantenna operates similarly as a receiver and a transmitter, that is, if the system emits light preferentially in a certain direction, it also enhances the local field more efficiently when the incident light is incoming from that same direction (and opposite orientation). Therefore, to ensure the maximum excitation enhancement of the quantum emitter, the applied external light source should propagate along the same direction of the maximum radiation of the nanoantennna, i.e., the maximum of the directivity, according to Figure 9 (with opposite orientation, and assuming again the same illumination and emission wavelength).

## 5 Conclusions

This work emphasizes the advantages of metallic nanostructures as effective enhancers of the photoluminescence emission from quantum emitters. The manipulation of size and shape of the nanostructures emerges as a crucial strategy in optimizing and fine-tuning enhancements for specific wavelength and directional preferences. Notably, we show that a large variety of metallic nanoantenna systems can strongly enhance the signal when their size is adequately tailored. Among the various nanoantennas studied, the rod gap nanoantenna exhibits the highest local electric field enhancement and photoluminescence. In contrast, systems based on spherical nanoparticles, while demonstrating lower PLEF, exhibit a high quantum yield across a broad spectrum of wavelengths and can be more easily fabricated in combination with self-assembly. Crescent gap nanoantennas, typically fabricated through lithography, display a large capability to enhance the electric field in the mid-IR spectral range, making them valuable building blocks for application in sensing and spectroscopy within this range. The disk gap and disk-on-mirror nanoantennas are easier to fabricate than the crescent and still offer large PLEF. This study provides valuable guidance in understanding and designing QE – nanoantenna hybrid systems with particular experimental conditions and available fabrication techniques, for specific applications. These applications could include biosensors, light-emitting diodes, and single-photon sources. While our results are based on idealized simulations that assume perfect emitter orientation and positioning, practical implementations may encounter challenges due to fabrication imperfections or misalignment of emitters. However, the guidelines presented here provide clear direction for optimizing emitter-antenna interactions and can serve as a strong foundation for experimental designs. Moreover, novel chemical techniques, such as DNA-based self-assembly, offer promising solutions for overcoming these limitations by precisely positioning quantum emitters within nanoantenna gaps.

Overall, this research serves as a comprehensive guide for researchers looking to use plasmonic nanostructures to enhance photoluminescence from quantum emitters, offering insights into future advancements in nanophotonic device design and applications of on-chip nanophotonic devices.

## Supplementary Material

Supplementary Material Details
