# Supplementary material for: An overview on plasmon-enhanced photoluminescence via metallic nanoantennas
Source: Nanophotonics. 2024 Nov 18;13(26):4771–94. doi: 10.1515/nanoph-2024-0463 (PMC11614590; doi:10.1515/nanoph-2024-0463)
Supplement: Supplementary file 1 — Supplementary Material Details [file j_nanoph-2024-0463_suppl_001.pdf]

Supporting Information

An Overview on Plasmon-enhanced Photoluminescence via Metallic Nanoantennas

José Luis Montaño-Priede,[†] Mario Zapata-Herrera,[†,‡] Ruben Esteban,[‡,†] Nerea Zabala,[¶,‡,†] and Javier Aizpurua[*,‡,¶,§]

†*Centro de Física de Materiales CFM-MPC (CSIC-UPV/EHU), Paseo Manuel de Lardizabal 5, 20018 Donostia, Spain*

‡*Donostia International Physics Center (DIPC), Paseo Manuel de Lardizabal 4, 20018 Donostia, Spain*

¶*Department of Electricity and Electronics, FCT-ZTF, UPV-EHU, Bilbao, 48080, Spain*

§*IKERBASQUE, Basque Foundation for Science, María Díaz de Haro 3, 48013 Bilbao, Spain*

E-mail: aizpurua@ehu.eus

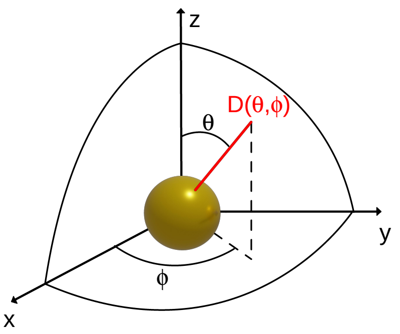

Figure S1: Scheme defining the polar (θ) and the azimuthal (ϕ) angles used in the definition of the directivity D of a nanoantenna.

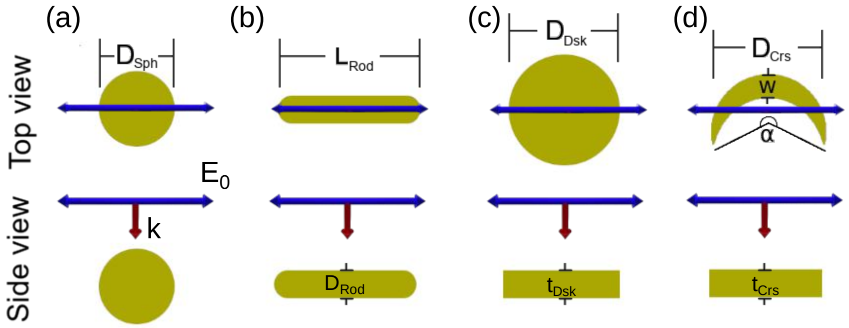

Figure S2: Geometrical parameters of the canonical nanostructures: (a) sphere, diameter in the range of 20 nm $\leq D_{\mathrm{Sph}} \leq$ 250 nm; (b) rod, length in the range 50 nm $\leq L_{\mathrm{Rod}} \leq$ 350 nm and fixed rod diameter of $D_{\mathrm{Rod}} = 25$ nm; (c) disk, diameter in the range of 100 nm $\leq D_{\mathrm{Dsk}} \leq$ 500 nm and fixed thickness of $t_{\mathrm{Dsk}} = 30$ nm; (d) crescent, diameter in the range of 100 nm $\leq D_{\mathrm{Crs}} \leq$ 500 nm, fixed thickness of $t_{\mathrm{Crs}} = 30$ nm, maximum width (central part) in the range of 21 nm $\leq \mathrm{w} \leq$ 105 nm, and fixed aperture angle of $\alpha = 211°$. The maximum width of the crescent (w) depends on D_{Crs} and we choose the value that corresponds to the material-implantation angle of $\theta = 30°$ during the fabrication process (see Ref. S1 for a detailed description and the dependence of D_{Crs} and w). Sharp edges are assumed for the disk and crescent in the vertical direction (side view), and the crescent tips have a curvature radius of ≈ 5 nm. The electric-field polarization (blue arrow) and the direction of propagation (red arrow) of the incoming light with respect to the nanostructures are indicated.

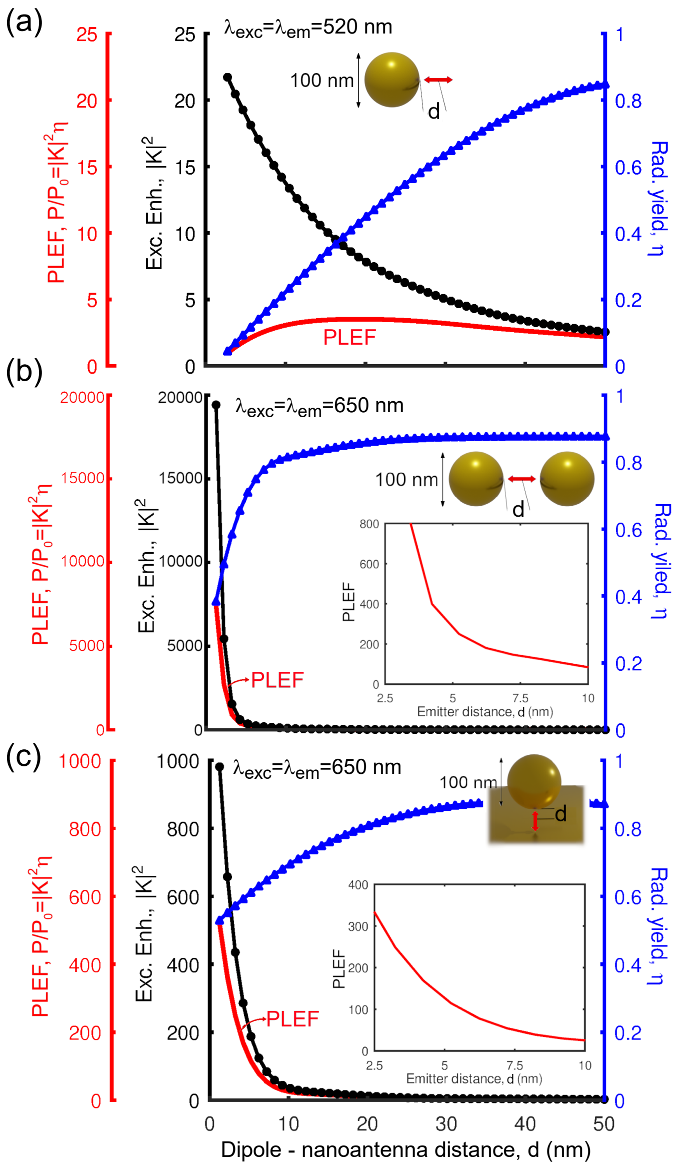

Figure S3: Excitation enhancement (black solid lines and circles), radiative yield (blue solid lines and triangles), and PLEF (red lines) of the three dipole - sphere nanoantenna configurations, (a) single, (b) gap, and (c) nanoparticle-on-mirror, as a function of the separation distance between the emitter and the Au sphere (diameter of D_{Sph} = 100 nm). The excitation wavelength (λ_{exc}) — equal to the emission wavelength (λ_{em}) — is (a) 520 nm, (b) 650 nm, and (c) 650 nm, which corresponds to the lowest-energy plasmonic mode of each nanoantenna.

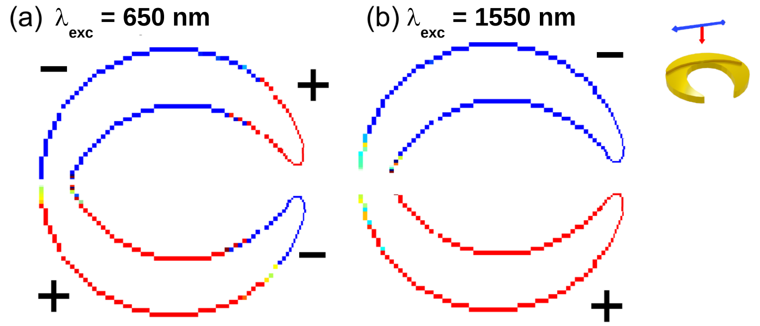

Figure S4: Charge distributions (ρ) of (a) the quadrupolar mode (at $\lambda_{\mathrm{exc}} = 650$ nm) and (b) the dipolar mode (at $\lambda_{\mathrm{exc}} = 1550$ nm) of the crescent gap nanoantenna (diameter of $D_{\mathrm{Crs}} = 160$ nm). It should be noted that the crescent-gap structure presents a raised joining region where the two crescent-shaped layers converge, resulting from the experimental growth process (see Ref. S1).

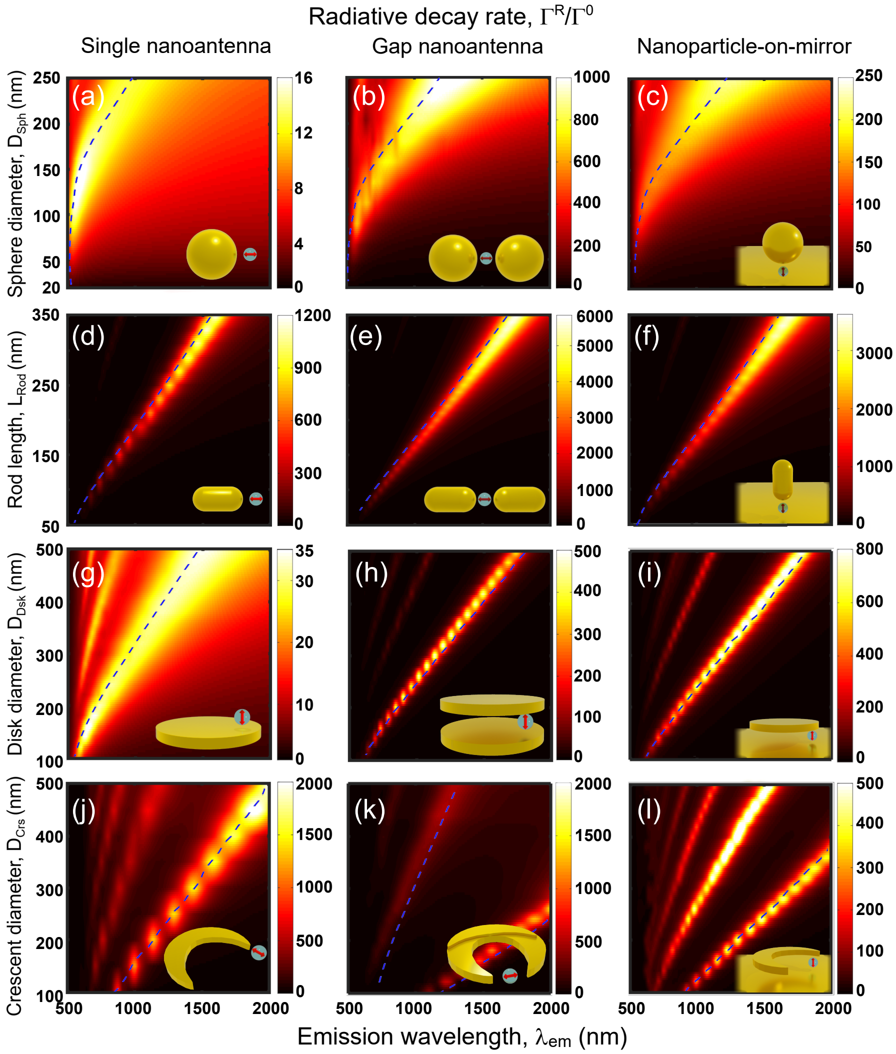

Figure S5: Maps of the radiative decay rate enhancement (Γ^R/Γ^0) of all the dipole - nanoantenna systems considered (see insets and Figure 2 in the main document) as a function of the nanostructure size and emission wavelength (λ_{em}). The size parameter changed and other dimensions of the nanoantennas, as well as the polarization of the excitation plane wave, are defined in Section 4.1 of the main document. The dipole is placed at d = 10 nm distance from the nanoantenna. The point-like dipole exciting the system is enclosed by a gray sphere in the insets to distinguish it from the background.

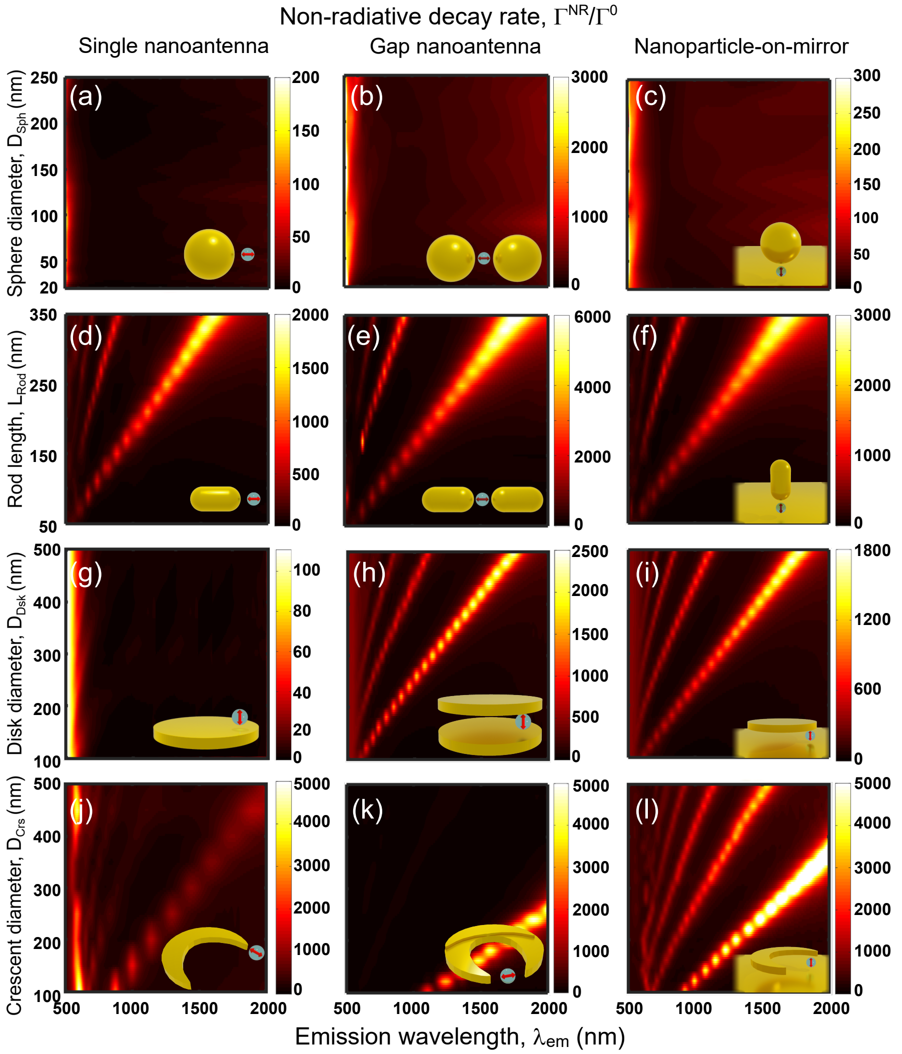

Figure S6: Maps of the non-radiative decay rate enhancement (Γ^{NR}/Γ^{0}) of the dipole - nanoantenna systems (see insets and Figure 2 in the main document) at the position as a function of the nanostructure size and emission wavelength (λ_{em}). The size parameter changed and other dimensions of the nanoantennas, as well as the polarization of the excitation plane wave are defined in Section 4.1 of the main document. The dipole is placed at d = 10 nm distance from the nanoantenna. The point-like dipole exciting the system is enclosed by a gray sphere in the insets to distinguish it from the background.

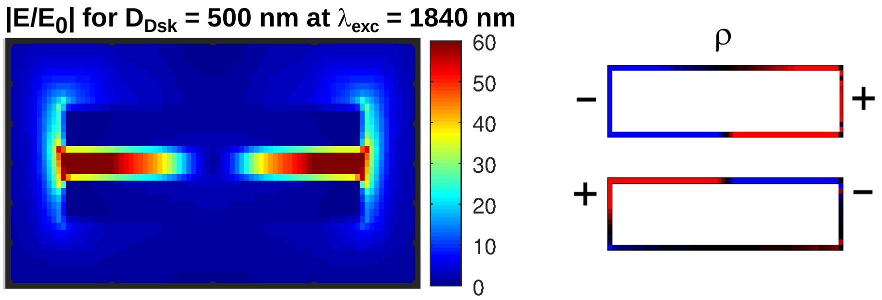

Figure S7: Normalized electric field ($|\mathbf{E}/\mathbf{E}_0|$) map and charge distribution (ρ) of a disk gap nanoantenna with $\mathrm{D_{Dsk}} = 500$ nm and $\lambda_{\mathrm{exc}} = 1840$ nm.

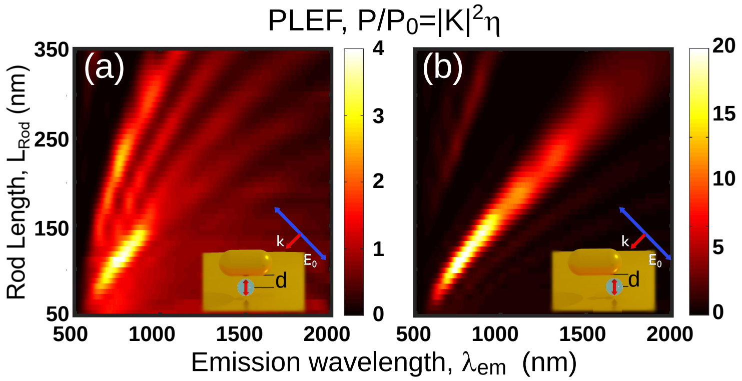

Figure S8: PLEF of the dipole interacting with the rod-on-mirror nanoantenna, as a function of the total rod length ($\mathrm{L_{Rod}}$) and emission wavelength (λ_{em}), when the rod is parallel to the mirror surface and the dipole is at (a) 0 nm and (b) $\mathrm{L_{Rod}}/2 - 5$ nm distance from the center to the tip of the rod. The gap separation between the rod and the substrate is d = 20 nm and the dipole is placed at the middle of this gap (10 nm from both tip and substrate), as indicated in the sketches in the insets. The point-like dipole that excites the system is enclosed by a gray sphere in these sketches to distinguish it from the background. The excitation wavelength is set to be equal to the emission wavelength $\lambda_{\mathrm{exc}} = \lambda_{\mathrm{em}}$.

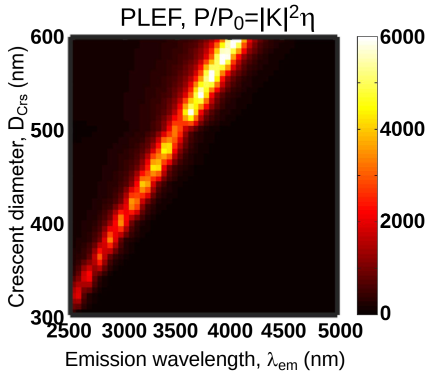

Figure S9: PLEF of the dipole - crescent gap nanoantenna system, as a function of the crescent diameter (D_{Crs}) and emission wavelength (λ_{em}), calculated in the mid-infrared spectral region (2500 nm $\leq \lambda_{em} \leq$ 5000 nm). The excitation wavelength is set to be equal to the emission wavelength $\lambda_{exc} = \lambda_{em}$.

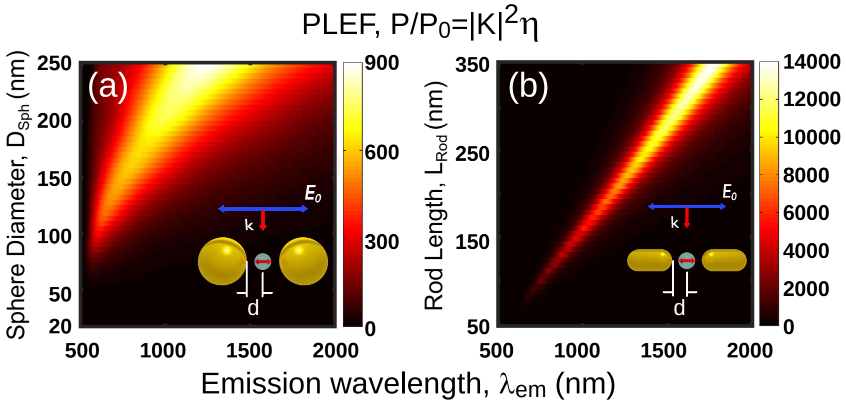

Figure S10: PLEF of the (a) dipole - sphere gap nanoantenna and (b) dipole - rod gap nanoantenna systems as a function of the nanostructure size (D_{Sph} and L_{Rod}, respectively) and emission wavelength (λ_{em}) for a distance d = 5 nm between the dipole and the nanoantenna. The excitation wavelength is set to be equal to the emission wavelength $\lambda_{exc} = \lambda_{em}$. The point-like dipole that excites the system is enclosed by a gray sphere in the insets to distinguish it from the background.

References

[S1] Rochholz, H.; Bocchio, N.; Kreiter, M. Tuning resonances on crescent-shaped noble-metal nanoparticles. *New J. Phys.* **2007**, *9*, 53.
